# Fruit shape loci *sun, ovate, fs8.1* and their interactions affect seed size and shape in tomato

**DOI:** 10.3389/fpls.2022.1091639

**Published:** 2023-01-12

**Authors:** Jie Chen, Bingqing Pan, Zixiong Li, Yue Xu, Xiaomeng Cao, Jingjing Jia, Huolin Shen, Liang Sun

**Affiliations:** ^1^ College of Horticulture, China Agricultural University, Beijing, China; ^2^ Beijing Key Laboratory of Growth and Developmental Regulation for Protected Vegetable Crops, Department of Vegetable Science, College of Horticulture, China Agricultural University, Beijing, China

**Keywords:** tomato, seed morphology, seed weight, cell number, cell size, transcriptome, phytohormone

## Abstract

Seed size and shape are not only critical for plant reproduction and dispersal, but also important agronomic traits. Tomato fruit shape loci *sun*, *ovate* and *fs8.1* regulate the morphology of fruit, flower, leaf and stem, and recently their functions in seed morphogenesis have also been noticed. However, mechanism underlying seed morphology variation has not been systematically investigated yet. Thus, using the near isogenic lines (NILs) harboring one, two or three of the fruit shape loci, histological, physiological and transcriptional bases of seed morphology change have been studied. *sun* and *ovate* showed potential abilities in decreasing seed size, whereas, *fs8.1* had a potential ability in increasing this parameter. Interactions between two loci and the interaction among three loci all led to significant decrease of seed size. All the loci significantly down-regulated seed shape index (SSI), except for *sun/fs8.1* double NIL, which resulted in the reductions in both seed length and width and finally led to a decreased trend of SSI. Histologically, seed morphological changes were mainly attributed to the cell number variations. Transcriptional and physiological analyses discovered that phytohormone-, cytoskeleton- as well as sugar transportation- and degradation-related genes were involved in the regulation of seed morphology by the fruit shape loci.

## 1 Introduction

Seed is an organ that connects two sexual life cycles of the flowering plant and is critical for plant reproduction and dispersal. Seed morphology, which mainly refers to the seed size and shape, is an important factor that impacts the above-mentioned processes. Seed size affects seed environmental adaptability as well as seed dispersal efficiency. Larger seeds accumulate more nutrients for germination and are more tolerant to abiotic stresses. Whereas, plants carrying smaller seeds tend to produce more seeds, which is conducive to the seed dispersal (Westoby et al., 2002; Moles et al., 2005). Seed shape also makes a great contribution to seed dispersal, especially for the wind spread seeds (Zhou et al., 2019). In another aspect, seed size is also an important agronomic trait not only for cereals but also for the horticultural crops, because some vegetable seeds are sold by weight and larger seeds often mean more income.

Seed morphology is controlled by the coordinated growth of the maternal (seed coat) and the zygote tissues (embryo and endosperm), which is regulated by multiple factors (Li et al., 2019). Among them, phytohormone-, sugar- and cell cycle-related processes play critical roles (Li et al., 2019; Ren et al., 2019; Xu et al., 2019; Wang et al., 2020; Zhao et al., 2022).

As to phytohormones, auxin is considered as a hormone with multiple functions which is involved in nearly all the events in plant life cycle, including seed development. In rice, a loss-of-function mutation of *TGW6*, the gene underlying a major grain weight quantitative trait loci (QTL), increased grain length and weight by delaying endosperm cellularization through affecting IAA (indole-3-acetic acid) production (Ishimaru et al., 2013). *AUXIN/INDOLE-3-ACETIC ACID* (*Aux/IAA*) and *AUXIN RESPONSE FACTOR* (*ARF*) which encode transcriptional repressor and transcription factor, respectively, are key components of the auxin signaling pathway (Weijers and Friml, 2009). The loss-of-function mutation of *AtARF2* in Arabidopsis resulted in the increase of seed size through promoting cell proliferation (Schruff et al., 2006). Meanwhile, in rice, *OsARF4* mediated the inhibition of grain size by *OsGSK5*/*OsSK41* (Hu et al., 2018). Additionally, *JcARF19* is the gene underlying a major QTL for seed length in the woody plant *Jatropha curcas* (Ye et al., 2014). In *Brassica napus*, a nature mutation of *BnARF18* decreased seed size through limiting the cell expansion in the silique wall (Liu et al., 2015a). Auxin polar distribution also takes part in the seed size control. A dominant mutation of *BIG GRAIN1* (*bg1-D*) changed the basipetal distribution of auxin and enhanced cell division and expansion in the spikelet hulls, which finally led to the enlargement of rice grain (Liu et al., 2015b). Cytokinin (CK) is another important phytohormone that impacts seed size. A wheat *TaCKX6-D1* gene, encoding a cytokinin oxidase/dehydrogenase (CKX), was associated with grain weight through association mapping (Zhang et al., 2012). Consistently, ectopic overexpression of Arabidopsis *AtCKX2* in tomato decreased of both seed size and number (Gan et al., 2022). In contrast, up-regulation of *AtCKX* in Arabidopsis resulted in the enlargement of seed size and the decrease of seed number (Werner et al., 2003). Gibberellins (GAs) have long been thought to play fundamental roles in seed development and germination, e.g., seed growth was significantly impacted in GA-deficient tomato mutant *ga-1* (Groot et al., 1987). Gibberellin 2-oxidase (GA2ox) catalyzes the bioactive GAs or their immediate precursors into the inactive forms and is important for controlling the GA level as well as keeping the homeostasis of this hormone (Hedden and Phillips, 2000). Overexpression of *JcGA2ox6*, a member of *GA2ox* family, in *Jatropha curcas* and Arabidopsis resulted in smaller fruits/shorter siliques and smaller seeds (Hu et al., 2017). Abscisic acid (ABA) is not only involved in the regulation of seed dormancy through antagonizing with gibberellins, but also plays vital roles in controlling seed growth as well as stimulating the biosynthesis of storage metabolites (Nambara and Marion-Poll, 2003; Cheng et al., 2014). In Arabidopsis, ABA-deficient mutant *aba2-1* produced larger seeds with an increased number of embryo cell (Cheng et al., 2014). In watermelon, knockout of *ClBG1*, which encodes a β-glucosidase and catalyzes the one-step hydrolysis of Glc-conjugated ABA, significantly reduced seed size and weight by decreasing cell number (Wang et al., 2021). Besides the above-mentioned hormones, brassinosteroid (BR) also regulates seed morphology by affecting the development of maternal tissue and the endosperm (Jiang et al., 2013; Li et al., 2019). In Arabidopsis and rice, seed length was repressed in the BR-deficient and BR-insensitive mutants (Jiang et al., 2013; Fang et al., 2016). In rice, BR was reported to regulate grain size by promoting cell enlargement in the spikelet hell (Fang et al., 2016; Zhou et al., 2017). However, a study in Arabidopsis suggested that BR may control seed size through influencing endosperm development and altered seed shape by impacting the maternal tissue (Jiang et al., 2013). Additionally, interactions among ABA, BR and CK have also been discovered to affect seed size, which may partially depend on their effects on impacting the downstream IKU pathway (Jiang et al., 2013; Li et al., 2013; Cheng et al., 2014). Sugar transportation and degradation affect the source-sink relationship which influences the seed development and eventually changes seed morphology (Lemoine et al., 2013). SWEET proteins play a crucial part in sugar translocation, and in Arabidopsis, rice and soybean, mutations of the *SWEET* genes impaired sucrose delivery from seed coat and endosperm to embryo and resulted in smaller, lighter and/or “wrinkled” seeds (Chen et al., 2015; Yang et al., 2018b; Wang et al., 2020). STP is another sugar transporter and involves in plant growth regulation (Wei et al., 2020). In Arabidopsis, products of *STP8* and *STP12* may contribute to the intake of sugar in the pollen tube and embryo (Rottmann et al., 2018). Sucrose synthase (SuSy) and invertase (INV) convert sucrose to starch for storage and later use. In rice, a loss-of-function mutation of *OsVIN2* resulted in decreased grain size by altering sugar metabolism (Xu et al., 2019).

Besides the above-mentioned factors, fruit shape genes are also important regulators of plant organ morphosis. So far, four major fruit shape loci have been identified, including *sun*, *ovate*, *sov1* and *fs8.1* (van der Knaap et al., 2014; Lazzaro et al., 2018; Wu et al., 2018). *sun* originates from a *Rider*-mediated retrotransposition, which enables *IQD12* (*SUN* gene, *Solyc10g079240*) to take the advantage of the *DEFL1*’s promoter and to express at a much higher level. The *sun* locus as well as overexpression of *SUN* all led to the elongation of fruit, cotyledon and leaflet, which could be attributed to the increased cell number in proximal-distal direction and the decreased cell number in medio-lateral direction. Members of IQD family have been confirmed to act as Ca^2+^-regulated scaffolds and are able to impact the arrangement of microtubules through recruiting CaM, KLCR1 (KINESIN LIGHT CHAIN-RELATED PROTEIN-1) and SPR2 to the microtubules (Abel et al., 2013; Burstenbinder et al., 2017b; Wendrich et al., 2018). In addition, interactions between auxin and *IQDs* have also been reported (Burstenbinder et al., 2017a; Wendrich et al., 2018). *OVATE* (*Solyc02g085500*) and *SlOFP20* (*Solyc10g076180*) all belong to the *OFP* gene family. *ovate*, a null mutation of *OVATE* gene, is resulted from a SNP which leads to the emergence of a pre-mature stop-codon. *ovate* changes the round-shaped fruit into elongated pear-shaped fruit by promoting cell division along the proximal-distal axis but simultaneously repressing cell proliferation along the medial-lateral direction at the proximal end. Overexpression of *OVATE* not only affected the shape of the reproductive organs, but also decreased plant height and leaf size (Liu et al., 2002; Wu, 2015; van der Knaap and Ostergaard, 2018). *Sov1* (*suppressor of ovate*) is attributed to a ~31-Kb deletion in the upstream of *SlOFP20*, which increases the gene’s expression and eventually leads to ovary cell number variation by influencing cell division pattern (Wu et al., 2018). At the molecular level, interactions among OFPs and TRMs (van der Knaap et al., 2014; Lazzaro et al., 2018; Wu et al., 2018) have been reported to impact the dynamic balance between cytoplasmic- and microtubular-localized OFP-TRM protein complexes, which may influence the assembling of the TTP (TON1-TRM-PP2A) complex as well as the organization of microtubule arrays and PPB formation, and thus cell division patterns and cell growth (Lazzaro et al., 2018). *fs8.1* has been fine-mapped to a 3.03-Mb region on chromosome 8 and twelve strong candidate genes, such as *PPR*, *ERECTA*, *GTL2-like* and *CKX*, have been predicted. The main effect of *fs8.1* is on the regulation of fruit shape and weight by increasing the cell number in proximal-distal direction (Sun et al., 2015).

Near-isogenic lines (NILs) are a powerful genetic tool not only for mapping QTLs but also for clarifying their functions (Zhu et al., 2015). Fruit shape NILs investigated in this study have been successfully used in the dissection of the synergistic and epistatic interactions among *sun*, *ovate* and *fs8.1* in affecting the fruit shape (Wu et al., 2015). In our previous study, seed size and shape variations were observed among the fruit shape NILs, however, systematic study has not been performed to illustrate the mechanism underlying this phenomenon yet. Therefore, in this study, effects of *sun*, *ovate* and *fs8.1* as well as their interactions on the regulation of seed size and shape were investigated at morphological, histological and transcriptional levels. Visual detection indicated that mature seed size and shape index were significantly decreased in the double and triple NILs. Developmental analysis suggested that the variations of seed size and shape could be observed as early as 8 dpa (days post anthesis). At the histological level, seed size variations were attributed to the changes of the seed coat area and embryo area, which were mainly caused by the cell number changes. Transcriptomic and physiological analyses disclosed that phytohormones and their related genes, sugar transportation and degradation-related genes as well as cytoskeleton-related genes were impacted by the fruit shape loci, which may be the reasons for the seed size and shape changes.

## 2 Materials and methods

### 2.1 Near isogenic lines

NILs in *Solanum pimpinellifolium* accession LA1589 background, of which the interval regions harbor one, two or three of the tomato fruit shape loci (*sun*, *ovate* and *fs8.1*), were investigated in this study. Even though the mutant alleles of the fruit shape genes were not caused by the loss-of-function mutations, the three loci were still represented by lowercase italics. Details about the interval regions and the morphological characteristics of the NILs were described in Wu et al., 2015. All eight NILs, including WT, *sun, ovate, fs8.1, sun+ovate* (*s*/*o*)*, sun+fs8.1* (*s*/*f*)*, ovate+fs8.1* (*o*/*f*)*, sun+ovate+fs8.1* (*s*/*o*/*f*), were grown in the greenhouse with standard fertilizer application and water supply at China Agricultural University in year 2021 and 2022. In each repeat, at least eight plants of each genotype were grown and all the investigated plants were placed randomly in the greenhouse.

### 2.2 Morphological and histological analyses

#### 2.2.1 Seed size, shape and weight measurements

Mature seeds were collected from red-ripe fruits of the NILs. In order to eliminate the effect of pollination efficiency on seed number and development, fruits developed from hand-pollinated flowers were selected for seed collection. Mature seeds were first treated with 9% HCl for 30 min and then were rinsed with tap-water for 10 min. After that, seeds were placed on paper towel and dried in room temperature for 2 weeks. This experiment was repeated three times, where seeds of replicates 1 and 2 were fixed on petri dishes using double-sides taps, whereas, seeds of replicate 3 were fixed on petri dishes using 1% agarose. After that, the petri dishes were scanned with the EPSON V600 scanner in 1200 dpi. Images were analyzed by Tomato Analyzer 4.0 software (http://oardc.osu.edu/vanderknaap/tomato_analyzer.php) and seed area, length, width and shape index were measured. The thousand seed weight was calculated from the weight of 200 shade-dried seeds. In each repeat, 1000 seeds were collected from at least 25 fruits which were harvested from at least 8 plants of each NIL.

#### 2.2.2 Seed development

Anthesis flowers were hand pollinated and tagged, and fruits developed from those flowers were harvested at four representative stages: 8, 10, 14 and 20 dpa according to Xiao et al., 2009. The developing seeds at each stage were separated from the young fruits under a stereo microscope with tweezers and then were immediately placed on 1% agarose gel in the petri dishes. Later, the petri dishes were scanned with the EPSON V600 scanner at 1200 dpi. Images were analyzed using Tomato Analyzer 4.0 software and seed area, length, width and shape index were measured. The experiment was repeated three times, and in each repeat, at each developmental stage, at least 100 developing seeds were collected from at least 5 fruits which were harvested from 8 plants of each NIL.

#### 2.2.3 Histological analysis of mature seeds

**Figure 1:**
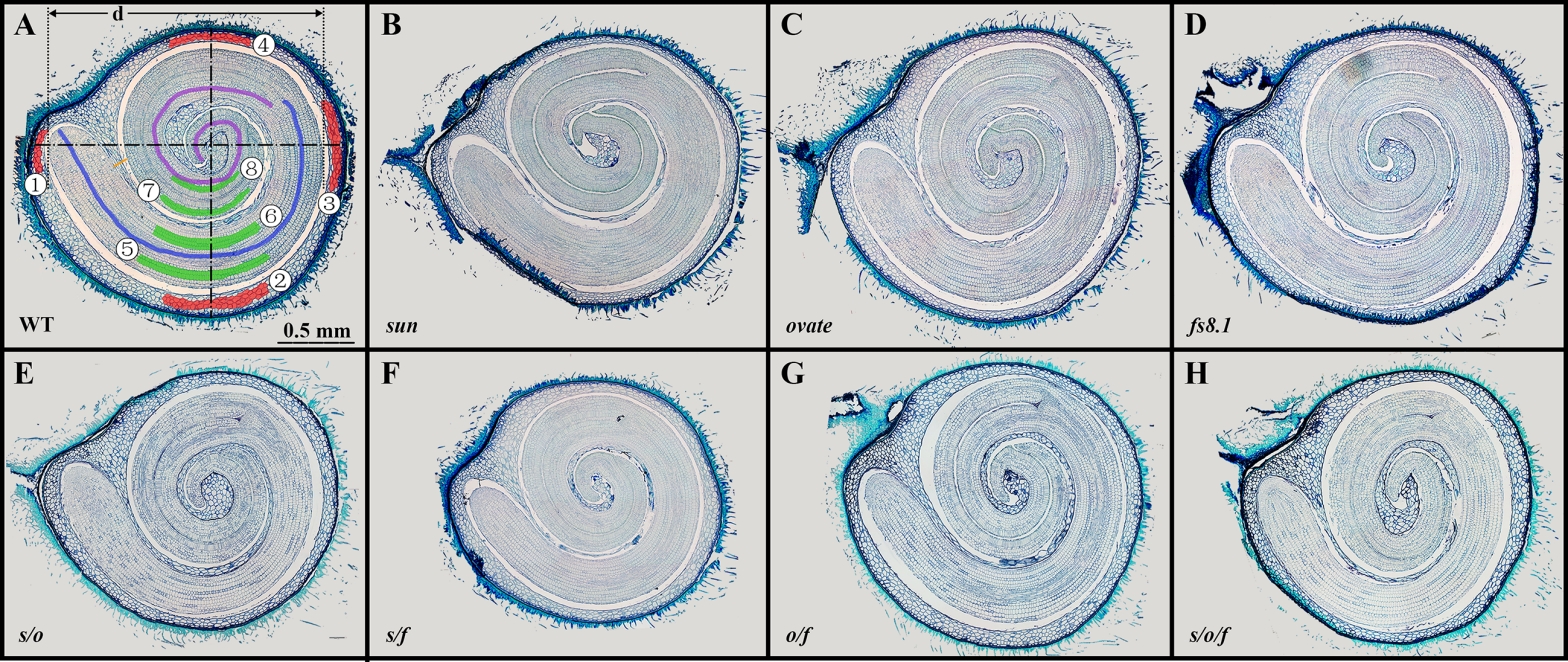
Paraffin sections of mature seeds of NILs WT **(A)**, *sun* **(B)**, *ovate* **(C)**, *fs8.1* **(D)***, s/o* **(E)***, s/f* **(F)***, o/f* **(G)** and *s/o/f* **(H)**. Detailed measurement methods are described in materials and methods.

Paraffin sections of mature seeds were made according to (Xiao et al., 2009) with some modifications. In order to soften the seed coat, mature seeds were immersed in 20% hydrofluoric acid for a week. After staining with toluidine blue, sections were photographed with the Olympus B73 microscope. Then, the images were measured using the ImageJ software. For each genotype, six seeds were embedded, and for each seed, at least 3 sections were made. As shown in **Figure 1A**, the orange bar represents the boundary of seed coat; the red areas represent where the seed coat cell size was measured; seed coat cell layer was counted along the dash-dotted lines; cell number of cotyledon and hypocotyl was measured along the purple curve and blue curve, respectively; the green area represents where the cell size of cotyledon and embryo was measured; seed coat cell number was represented by the total number of seed coat cells in the second layer along abaxial direction; distal and body cell layer and size=(②+③+④)/3; cotyledon cell size=(⑦+⑧)/2; hypocotyl cell size=(⑤+⑥)/2;embryo crimp ratio=d/embryo length.

### 2.3 Statistical analysis

Analysis of variance (ANOVA) and Turkey’s mean separation tests were performed in this paper using SPSS software. Significant effects and interactions were shown by the p-values computed from the F ratio in ANOVA. Visualization of the results were conducted using GraphPad.

### 2.4 RNA-Seq library construction, sequencing and analysis

Sixty to eighty developing seeds were dissected from at least three fruits of each NIL at 8 dpa and immediately frozen in liquid nitrogen. Total RNA extract, library construction and sequencing were performed by Biomarker Technologies Corporation (Beijing, China) on a GAII platform (Illumina). Preliminary data analysis, including short sequences (reads) filtering, alignment, and FPKM calculation, was performed in BMKCloud (www.biocloud.net). Since very few genes were statistically significant when using DESeq2 method, genes that met the following conditions were considered as the differentially expressed genes (DEGs) and selected for the Venn diagram analysis. (i) the average value of a gene’s FPKM must be ≥2 in any of the eight NILs; (ii) the ratio of FPKM value of a gene (any of the seven mutant NILs/WT) must be ≥1.50 or ≤0.67. Venn diagram analyses were conducted using Venny 2.0.2 software (https://bioinfogp.cnb.csic.es/tools/venny/index2.0.2.html). Heatmaps were drawn *via* using the TBtools software.

### 2.5 Quantitative real-time PCR analysis

For verifying the RNA-seq results, quantitative real-time PCR (qRT-PCR) was performed to clarify the expression patterns of some of the interested genes, including *DWF1* (*Solyc02g069490*), *2ODD* (*Solyc02g062490*), *GRP13* (*Solyc06g007920*), *GA2ox2* (*Solyc10g007560*), *NCED3* (*Solyc07g056570*), *VI2* (*Solyc08g079080*), *SWEET14* (*Solyc03g097560*) and *WLIM1* (*Solyc03g114000*). Total RNA used for RNA-seq analysis was reverse-transcribed into cDNA using a PrimeScript RT reagent kit (Takara, Dalian, Liaoning, China). Primers were designed using Primer5 software and listed in **Supplementary Table S2**. *SAND* (*Solyc03g115810*) was selected as the internal control gene according to Expositó-Rodríguez et al. (2008). 2^-ΔΔCt^ method was used to calculate relative expression values. For each gene, three technical repeats were performed.

### 2.6 Phytohormone analysis

Phytohormones were detected by MetWare (http://www.metware.cn/) based on the AB Sciex QTRAP 6500 LC-MS/MS platform. The hormones analyzed were: indole-3-acetic acid (IAA), abscisic acid (ABA), cytokinin (CK), gibberellins (GAs), brassinosteroid (BR) and ethylene (ETH). The significance of the effect of a locus on the accumulation of a certain hormone and interactions between loci were calculated by ANOVA. Paired Student’s t-tests were performed to compare the group means when the effect was significant. The effects of each locus were tested in the WT and mutant backgrounds.

## 3 Results

### 3.1 Fruit shape loci and their interactions impacted tomato seed size and shape

**Figure 2:**
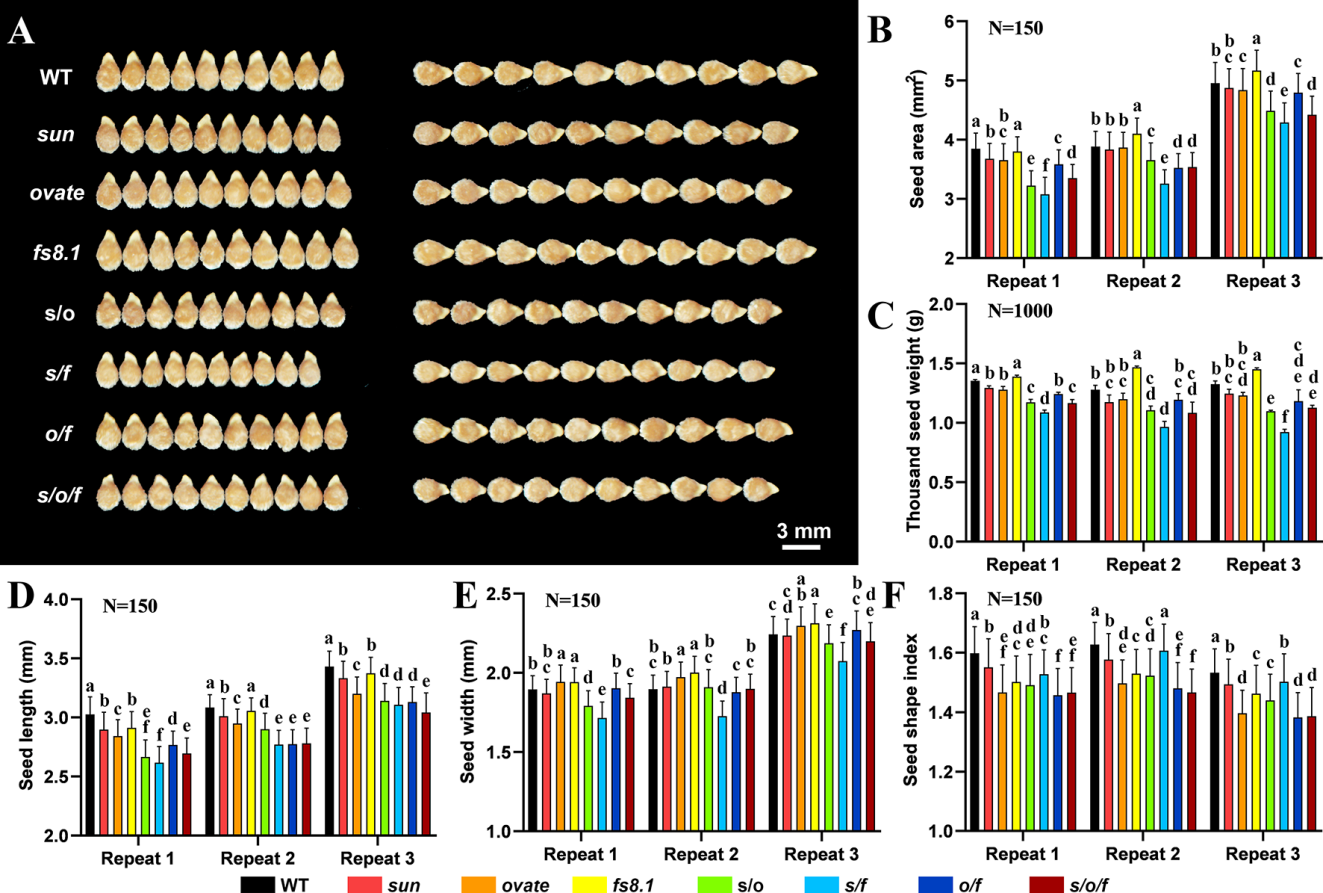
Seed size and shape variations of fruit shape NILs. **(A)** Mature seeds. **(B)** Seed area. **(C)** Thousand seed weight. **(D)** Seed length. **(E)** Seed width. **(F)** Seed shape index. “N” stands for the number of seeds that was analyzed for each genotype in each replication. Pairwise comparisons between the NILs were done with ANOVA and means were separated with Turkey’s test α < 0.05. Small alphabet letters above the bars indicate the significance of the HSD test.

Mature seeds collected from tomato fruit shape NILs were investigated visually (**Figure 2A**). Compared with that in WT, seed size was significantly decreased in the double and triple NILs, meanwhile, decreased trends of seed size were recorded in NILs *sun* and *ovate*; in contrast, an increased trend of seed size was discovered in *fs8.1* NIL (**Figure 2B**). Consistent with that, similar variation patterns were discovered for thousand seed weight (**Figure 2C**). As to seed length, it was significantly down-regulated in all the mutant NILs, except for *fs8.1*, in which significant decreases were only recorded in two of the three repeats (**Figure 2D**). In regard to seed width, it was significantly up-regulated in NILs *ovate* and *fs8.1* but was down-regulated in *s*/*f* NIL; meanwhile, decreased trends of seed width were observed in NILs *s*/*o* and *s*/*o*/*f*; different from what is mentioned above, no significant difference of seed width was found among NILs WT, *sun* and *o*/*f* (**Figure 2E**). In the case of seed shape index (SSI) which is determined by both the seed length and width, it was significantly reduced in all the mutant NILs, except for the *s/f* NIL, in which significant decreases were only recorded in two of the three repeats (**Figure 2F**).

In order to evaluate the effect of *sun*, *ovate* and *fs8.1* on the above-mentioned parameters as well as clarify the interactions among the three loci, contrasts and three-way ANOVA were performed. Based on the contrast’s results, *ovate* showed the greatest effect on seed length, meanwhile, all three fruit shape loci showed significant effects on the seed shape index (**Table 1**). Epistasis was discovered in NILs *s/o*, *s/f* and *s/o/f* for at least one studied parameter. Particularly, interaction between *sun* and *ovate* significantly impacted seed size, thousand weight and length; meanwhile, interaction between *sun* and *fs8.1* significantly affected seed size, thousand weight, width and shape index; moreover, interaction among the three loci influenced all the studied parameters (**Table 2**).

**Table 1:** Comparisons of the effects of *sun, ovate* and *fs8.1* on seed area, thousand seed weight, seed length, seed width and seed shape index.

	Contrast coefficients								Pr (>|t|)																
Comparison	WT	*sun*	*ovate*	*fs8.1*	*s/o*	*s/f*	*o/f*	*s/o/f*	SA			TSW				SL			SW				SSI		
Mutants vs. WT	-7	1	1	1	1	1	1	1	**<0.0001**	**<0.0001**	**<0.0001**	**<0.0001**	**0.0035**	**<0.0001**		**<0.0001**	**<0.0001**	**<0.0001**	0.0594	0.7185	<0.0001		**<0.0001**	**<0.0001**	**<0.0001**
*sun* vs. WT	-1	1	0	0	0	0	0	0	0.0197	0.0896	<0.0001	**0.0250**	**0.0246**	**0.0025**		**<0.0001**	**<0.0001**	**<0.0001**	0.5631	0.1612	0.0246		**<0.0001**	**<0.0001**	**<0.0001**
*ovate* vs. WT	-1	0	1	0	0	0	0	0	0.0008	0.5267	<0.0001	0.0100	0.0750	0.0004		**<0.0001**	**<0.0001**	**<0.0001**	**<0.0001**	**<0.0001**	**<0.0001**		**<0.0001**	**<0.0001**	**<0.0001**
*fs8.1* vs. WT	-1	0	0	1	0	0	0	0	<0.0001	<0.0001	0.1121	0.0015	0.0004	0.0592		0.0001	0.0553	<0.0001	**<0.0001**	**<0.0001**	**<0.0001**		**<0.0001**	**<0.0001**	**<0.0001**
*sun* vs. *ovate*	0	1	-1	0	0	0	0	0	0.3115	0.2868	0.4329	0.6591	0.5718	0.3734		**<0.0001**	**<0.0001**	**0.0006**	**<0.0001**	**<0.0001**	**<0.0001**		**<0.0001**	**<0.0001**	**<0.0001**
*sun* vs. *fs8.1*	0	1	0	-1	0	0	0	0	**<0.0001**	**<0.0001**	**<0.0001**	**<0.0001**	**<0.0001**	**<0.0001**		0.0069	0.0020	0.3276	**<0.0001**	**<0.0001**	**<0.0001**		**0.0007**	**<0.0001**	**<0.0001**
*ovate* vs. *fs8.1*	0	0	1	-1	0	0	0	0	**<0.0001**	**<0.0001**	**<0.0001**	**<0.0001**	**<0.0001**	**<0.0001**		**<0.0001**	**<0.0001**	**<0.0001**	0.1694	0.0100	0.9949		**<0.0001**	**0.0008**	**0.0007**

SA, seed area; TSW, thousand seed weight; SL, seed length; SW, seed weight; SSI, seed shape index. Bold-type indicates significant difference in the statistical analysis. Bold-type indicates significant difference in the statistical analysis.

**Table 2:** Effects and interactions of *sun*, *ovate* and *fs8.1* on the size and shape attributes of mature seeds.

	Pr > *F*						
Attribute	*sun*	*ovate*	*fs8.1*	*sun × ovate*	*sun × fs8.1*	*ovate × fs8.1*	*sun × ovate × fs8.1*
Seed area	**<0.0001**	**<0.0001**	**<0.0001**	**0.0008**	**<0.0001**	0.0002	**<0.0001**
	**<0.0001**	**<0.0001**	**<0.0001**	**<0.0001**	**<0.0001**	0.0938	**<0.0001**
	**<0.0001**	**<0.0001**	**<0.0001**	**0.0001**	**<0.0001**	<0.0001	**<0.0001**
Thousand seed weight	**<0.0001**	**0.0003**	0.0042	**<0.0001**	**<0.0001**	0.0134	**<0.0001**
	**<0.0001**	**0.0025**	0.5793	**0.0002**	**0.0002**	0.9670	**0.0004**
	**<0.0001**	**<0.0001**	<0.0001	**0.0001**	**<0.0001**	0.0013	**<0.0001**
Seed length	**<0.0001**	**<0.0001**	**<0.0001**	**<0.0001**	<0.0001	0.0001	**<0.0001**
	**<0.0001**	**<0.0001**	**<0.0001**	**<0.0001**	<0.0001	0.3536	**<0.0001**
	**<0.0001**	**<0.0001**	**<0.0001**	**<0.0001**	0.0530	<0.0001	**<0.0001**
Seed width	**<0.0001**	**0.0004**	**<0.0001**	0.0077	**<0.0001**	0.0015	**<0.0001**
	**<0.0001**	**0.0000**	**<0.0001**	<0.0001	**<0.0001**	0.2841	**<0.0001**
	**<0.0001**	**0.0107**	**<0.0001**	0.0697	**<0.0001**	<0.0001	**<0.0001**
Seed shape index	0.0096	**<0.0001**	**<0.0001**	0.0109	**0.0300**	0.8044	**<0.0001**
	0.0463	**<0.0001**	**<0.0001**	0.4726	**<0.0001**	0.7523	**<0.0001**
	0.5794	**<0.0001**	**<0.0001**	0.0095	**0.0070**	0.0001	**<0.0001**

Significant effects and interactions were shown by the p-values computed from the F ratio in ANOVA. Bold-type indicates significant difference in the statistical analysis.

### 3.2 Seed size and shape variations were mainly attributed to the cell number changes

In order to illustrate the histological bases for the seed size and shape variations among the tomato fruit shape NILs, paraffin sections of mature seeds were made and seed structure, seed cell number and cell size were analyzed (**Figure 1**; **Table 3**). In terms of seed structure, seed coat area, cavity area, embryo area, embryo length and embryo crimp ratio (ratio of embryo diameter (*d*) to length) were investigated (**Figure 1A**). Except for the embryo crimp ratio, all the above-mentioned parameters were significantly correlated with seed size (**Supplementary Table S1**), suggesting seed size variations were caused by the changes of seed coat area, cavity area as well as embryo area and length. Particularly, consistent with what observed in seed size, compared with those in WT, seed coat area, cavity area, embryo area and embryo length were not significantly changed in *sun* and *ovate* NILs (**Table 3**). Whereas, in *fs8.1* NIL, of which the seed size showed clearly increased trend, cavity area was significantly increased, indicating the increase of seed size in this NIL was mainly attributed to the size change of cavity area (**Table 3**). In contrast to the single NILs, seed coat area, cavity area as well as embryo area and length were all significantly decreased in *s*/*o* and *s*/*f* NILs (**Table 3**), suggesting those tissues/structures determined seed size of those two NILs; meanwhile, only seed coat area was significantly reduced in *o*/*f* NIL (**Table 3**), which may be the major reason for the smaller seed size in this NIL; in the *s*/*o*/*f* NIL, seed coat area and embryo area were significantly downregulated (**Table 3**), implying the seed size variation in this NIL was mainly due to the changes of those two tissues.

**Table 3:** Morphological and histological analyses of internal organs of mature seeds in *sun*, *ovate* and *fs8.1* NILs. .

	Seed coat area (mm^2^)			Cavity area (mm^2^)			Embryo area (mm^2^)			Embryo length (mm)				Embryo crimp ratio		

WT	0.76±0.02	ab		0.45±0.02	b		1.72±0.06	ab		5.48±0.08	a			38.28%±0.25%		a
*sun*	0.73±0.02	b		0.45±0.02	b		1.63±0.04	b		5.33±0.24	ab			38.64%±0.29%		a
*ovate*	0.73±0.02	b		0.48±0.02	ab		1.65±0.05	ab		5.48±0.26	a			36.92%±0.21%		b
*fs8.1*	0.79±0.01	a		0.52±0.02	a		1.80±0.04	a		5.61±0.15	a			38.11%±0.35%		a
*s/o*	0.64±0.01	cd		0.39±0.01	c		1.40±0.03	c		5.02±0.14	bc			36.67%±0.39%		bc
*s/f*	0.60±0.02	d		0.37±0.01	c		1.22±0.04	d		4.68±0.15	c			38.41%±0.26%		a
*o/f*	0.66±0.03	c		0.45±0.02	b		1.65±0.07	ab		5.48±0.19	a			35.92%±0.79%		bc
*s/o/f*	0.67±0.02	c		0.45±0.03	b		1.38±0.03	cd		5.32±0.07	ab			35.74%±0.30%		c
	Seed coat cell number			Seed coat cell layer						Seed coat cell size (μm^2^)						
				distal and body direction			proximal direction			distal and body direction				proximal direction		
WT	212.33±6.74		ab	5.00±0.44		ab	5.44±1.24	a		687.36±55.03		b		562.20±28.95	a	
*sun*	199.25±6.30		cd	4.86±0.44		ab	5.83±0.72	a		751.75±69.30		ab		550.96±22.92	a	
*ovate*	200.67±1.76		bc	4.83±0.56		ab	5.38±1.19	a		728.76±90.64		ab		592.77±39.12	a	
*fs8.1*	217.75±2.53		a	5.17±0.44		a	5.63±0.74	a		742.06±25.87		ab		594.07±26.27	a	
*s/o*	189.17±4.04		de	4.72±0.41		ab	6.56±1.33	a		692.45±66.55		b		579.15±52.26	a	
*s/f*	178.00±3.65		e	4.40±0.34		b	5.20±1.03	a		811.53±65.33		a		590.86±45.86	a	
*o/f*	198.00±6.00		cd	4.87±0.51		ab	5.20±0.84	a		739.81±75.18		ab		535.80±30.12	a	
*s/o/f*	187.25±4.33		de	4.63±0.53		ab	6.00±1.12	a		749.52±30.30		ab		560.78±38.33	a	

	Cotyledon cell number			Hypocotyl cell number			Cotyledon cell size (μm^2^)			Hypocotyl cell size (μm^2^)						
WT	147.00±2.45		b	174.25±3.50		d	269.32±25.56		a	405.95±55.77			a			
*sun*	150.00±5.10		b	177.25±2.50		cd	275.25±21.42		a	392.42±61.79			a			
*ovate*	163.25±5.38		a	186.00±4.32		abc	282.67±26.01		a	407.44±32.36			a			
*fs8.1*	157.00±6.06		ab	190.00±4.40		a	282.48±34.32		a	409.55±37.52			a			
*s/o*	147.25±6.40		b	177.75±2.63		bcd	233.62±19.96		a	406.25±40.09			a			
*s/f*	127.00±2.94		c	160.50±5.00		e	231.83±34.03		a	354.43±39.77			a			
*o/f*	153.50±5.07		ab	186.75±3.10		ab	267.57±14.89		a	427.02±42.21			a			
*s/o/f*	147.25±5.85		b	185.50±5.26		abc	246.55±16.38		a	406.61±31.90			a			

Pairwise comparisons between the NILs were done with ANOVA and means were separated with Turkey’s test α < 0.05.

Organ size variation is generally resulted from the alterations of cell division and/or expansion patterns. In this study, compared with that in WT, seed coat cell number in proximal-distal direction (P-D) was significantly decreased in *sun*, double and triple NILs; whereas, seed coat cell layer was not significantly changed in any of the mutant NILs; as to the seed coat cell size, it was only significantly increased in the distal and body (DB) areas in *s*/*f* NILs, but was not changed in the proximal area (**Table 3**). In regard to the embryo, cotyledon cell number was significantly up- and down-regulated in *ovate* and *s*/*f* NILs, respectively; meanwhile, hypocotyl cell number was significantly increased in *ovate*, *fs8.1*, *o*/*f* and *s*/*o*/*f* NILs, but was decreased in *s*/*f* NILs; as to the embryo cell size, no significant change was observed in cotyledon and hypocotyl (**Table 3**).

In the aspect of seed shape, it was not only determined by cell number and size, but also controlled by the embryo crimp pattern. However, since cell size of embryo was not affected by tomato fruit shape loci (**Table 3**) and the significant change of seed coat cell size in the DB of *s*/*f* NIL was opposite to variation trends of seed length and width (**Table 3**; **Figures 2D, E**), it can be presumed that cell number may played a more important role in the regulation of seed shape than cell size. Taking together, in this study, tomato fruit shape loci regulated seed size and shape mainly through impacting the cell number of seed coat and embryo.

### 3.3 Seed size and shape occurred at early developmental stages

**Figure 3:**
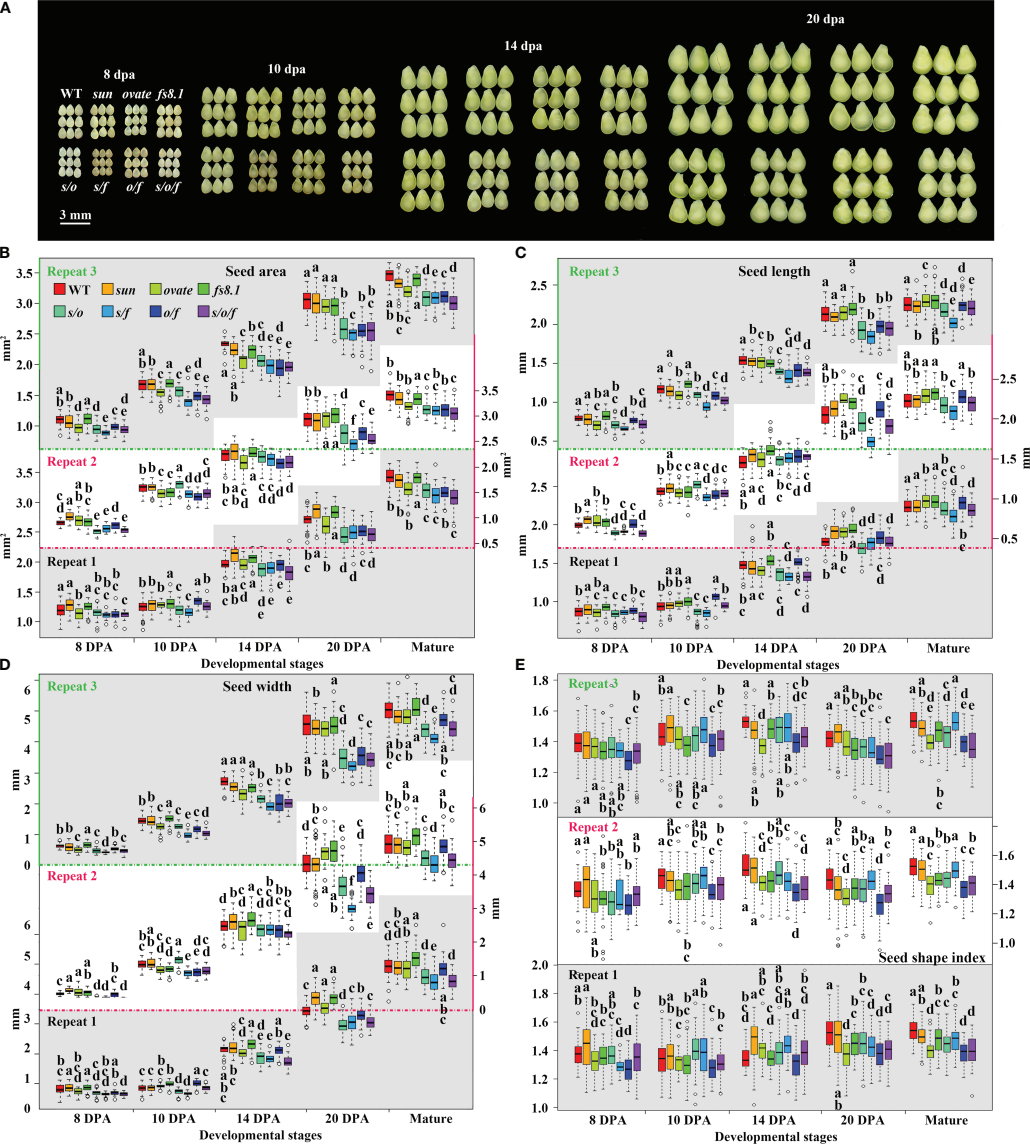
Comparisons of seed size and shape at different developmental stages in fruit shape NILs. **(A)** Representative seeds from eight genotypes at 8, 10, 14 and 20 dpa. **(B)** Changes in seed area from 8 dpa to 20 dpa. **(C)** Changes in seed length from 8 dpa to 20 dpa. **(D)** Changes in seed width from 8 dpa to 20 dpa. **(E)** Changes in seed shape index from 8 dpa to 20 dpa. Statistical analyses were performed using Turkey HSD tests at 0.05. Small alphabet letters above or below the boxes indicate the significance of the HSD test.

For clarifying the effects of fruit shape loci on seed development, developing seeds were separated from young fruits at 8, 10, 14 and 20 dpa (**Figure 3A**). Similar development trends were observed in all the NILs for seed size, length and width, which looked like equilibrium curves (**Figures 3B–D**; **Supplementary Figure S1**). In contrast, N-shaped development curves were recorded in all eight NILs for SSI (**Figure 3E**; **Supplementary Figure S1**). At each stage, the effects of fruit shape loci on seed size and shape were similar to those at the mature stage, although statistical significance was not always found between each mutant NIL and WT NIL in every repeat (**Figures 3A–E**; **Supplementary Figure S2**). In another aspect, at 8 dpa, *s*/*o*, *s*/*f* and *s*/*o*/*f* significantly decreased seed size in all the repeats, and meanwhile, *o*/*f* significantly down-regulated SSI in all the repeats (**Figures 3B–E**; **Supplementary Figure S2**), suggesting fruit shape loci and their interactions played critical roles in the regulation of seed size and shape at no later than 8 dpa.

### 3.4 Phytohormone levels were impacted in developing seeds

**Figure 4:**
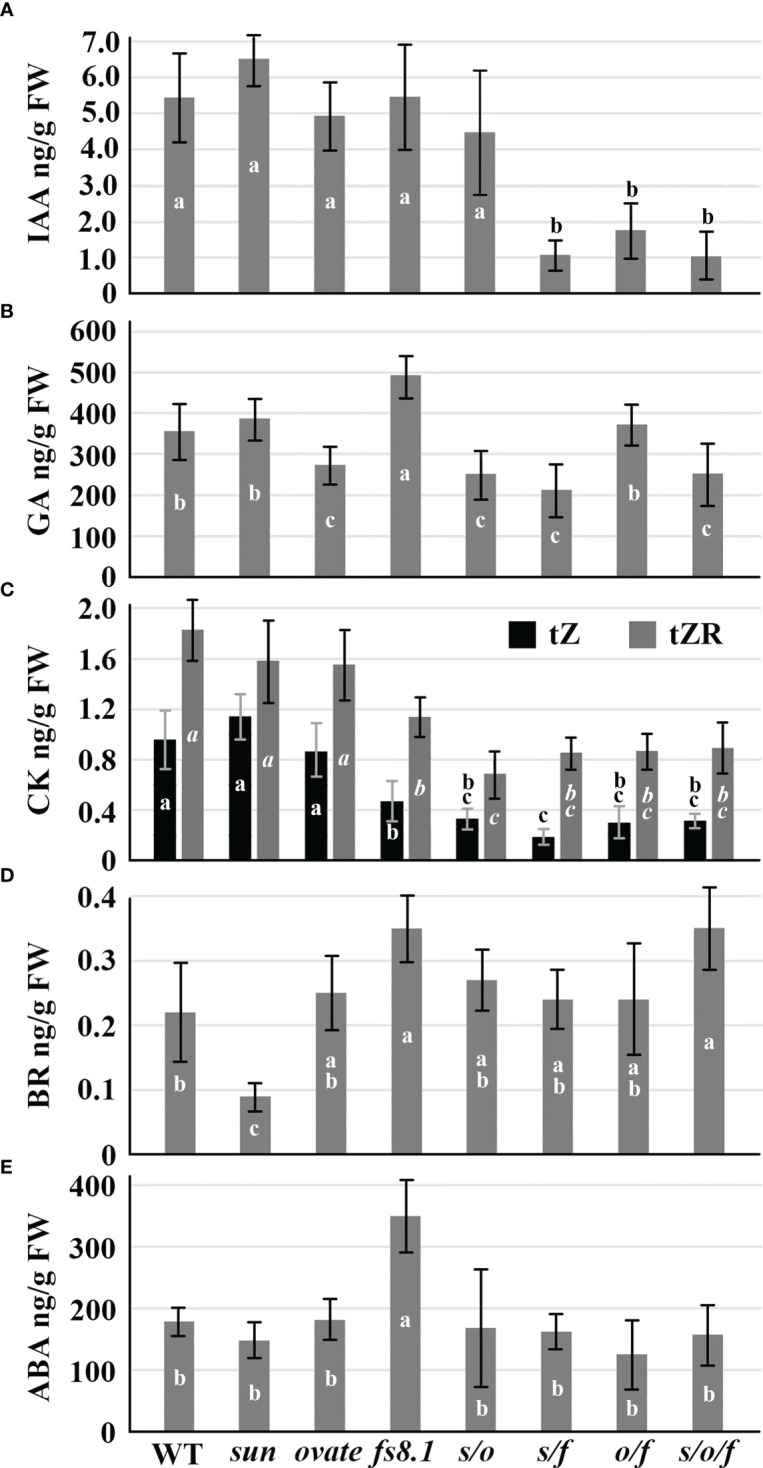
IAA **(A)**, GA **(B)**, CK **(C)**, BR **(D)** and ABA **(E)** contents of developing seeds at 8 dpa in fruit shape NILs. Small alphabet letters in the bars indicate the significance of the HSD test.

Compared with WT, IAA was significantly decreased in NILs *s/f*, *o/f* and *s/o/f*; total GA content was significantly up-regulated in *fs8.1* NIL, but was down-regulated in NILs *ovate*, *s/o*, *o/f* and *s/o/f*; except for NILs *sun* and *ovate*, levels of *trans*-zeatin and *trans*-zeatin riboside were reduced in all mutant NILs; BR content was significantly decreased in *sun* NIL, but was increased in NILs *fs8.1* and *s/o/f*; the accumulation of ABA was only enhanced in *fs8.1* NIL (**Figure 4**).

### 3.5 Fruit shape loci regulated seed morphology mainly through affecting the phytohormone-, sugar- and cytoskeleton-related pathways at the transcriptional level

**Figure 5:**
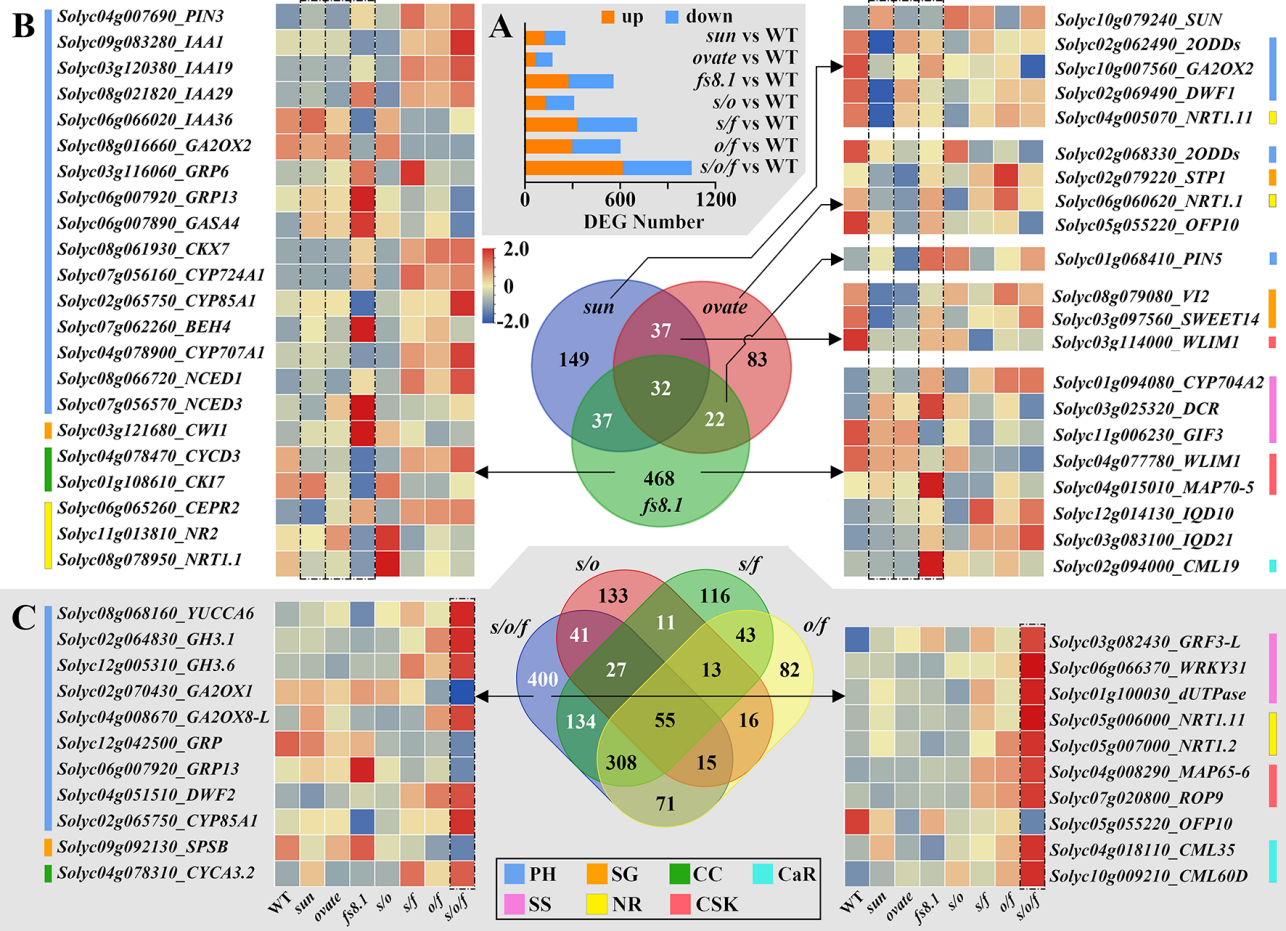
Number of DEGs and Venn diagram analyses of transcriptomes of seeds in fruit shape NILs. **(A)** Differentially expressed genes (DEGs) number at 8 dpa. **(B)** Venn diagram of *sun*, *ovate* and *fs8.1* NILs. **(C)** Venn diagram of double and triple NILs. PH, plant hormone-related genes; SG, suger-related genes; CC, cyclin; CaR, calcium-related genes; SS, seed size-related genes; NR, nitrate-related genes; CSK, cytoskeleton-related genes.

For exploring the regulatory mechanisms of fruit shape loci on seed size and shape at the transcriptional level, RNA-seq analysis was performed with seeds collected from 8-dpa fruits. Comparisons were conducted between each mutant NIL and WT NIL, and DEGs were identified. *sun*, *ovate*, *fs8.1*, *s*/*o*, *s*/*f*, *o*/*f* and *s*/*o*/*f* led to 255 (up-/down-regulated (hereafter u/d) =124/131), 174 (u/d=69/105), 559 (u/d=274/285), 311 (u/d=133/178), 707 (u/d=329/378), 603 (u/d=298/305) and 1051 (u/d=617/434) DEGs, respectively (**Figure 5A**). Enrichment and pathway analyses indicated that there were common pathways or function classes among *sun*, *ovate* and *fs8.1*, and there were additive and epistatic effects among the three loci (**Supplementary Figures S3-S6**). Transcription factor enrichment revealed that B3, AP2 and MYB were the most common families that were discovered in the developing seeds (**Supplementary Figure S7**).

**Figure 6:**
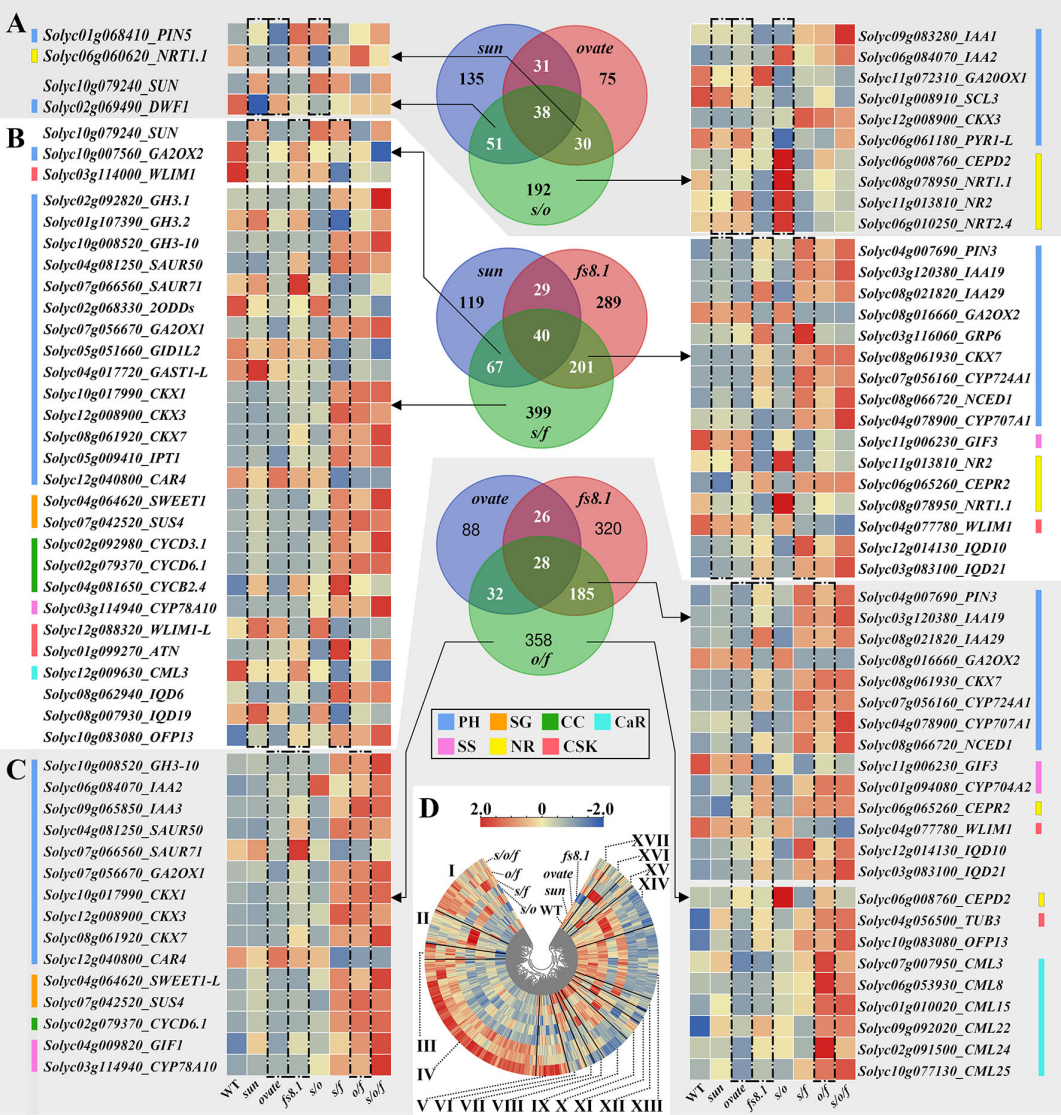
Venn diagram analyses of fruit shape NILs and the integrated expression patterns of the interested DEGs. **(A)** Venn diagram of *sun*, *ovate* and *s/o* NILs. **(B)** Venn diagram of *sun*, *fs8.1* and *s/f* NILs. **(C)** Venn diagram of *ovate*, *fs8.1* and *o/f* NILs. **(D)** Expression clustering of all the DEGs in fruit shape NILs. PH, plant hormone-related genes; SG, sugar-related genes; CC, cyclin; CaR, calcium-related genes; SS, seed size-related genes; NR, nitrate-related genes; CSK, cytoskeleton-related genes.

For further dissecting the transcriptional bases of each fruit shape locus and the interactions between different loci, Venn diagram analyses were performed (**Figures 5**, **6**). In the Venn diagram of NILs *sun*, *ovate* and *fs8.1*, there were 149, 83 and 468 DEGs unique to NILs *sun*, *ovate* and *fs8.1*, respectively; meanwhile, 37, 37 and 22 DEGs were shared between NILs *sun* and *ovate*, NILs *sun* and *fs8.1*, and NILs *ovate* and *fs8.1*, respectively; additionally, 32 DEGs were shared by NILs *sun*, *ovate* and *fs8.1* (**Figure 5B**). Based on genes’ annotations, 43 interested DEGs were screened out (**Figure 5B**). Among them, *SUN* was highly expressed in the NILs harboring *sun*; *CWI1* (encoding a cell wall invertase which is involved in sucrose degradation), *GRP13* (encoding a gibberellin-regulated protein), *GASA4* (encoding a gibberellin-regulated protein), *DCR* (encoding a BAHD acyltransferase whose homolog controls rice grain size by modulating brassinosteroid homeostasis (Feng et al., 2016)), *BEH4* (encoding BES1/BZR1 homolog protein 4 which takes part in BR signal transduction), *CML19* (encoding a calmodulin-like protein which mediates multiple reactions involved in regulation of plant growth), *NCED3* (encoding a 9-cis-epoxycarotenoid dioxygenase which plays a part in ABA biosynthesis) and *MAP70-5* (encoding a microtubule-associated protein 70-5 which may be involved in the regulation of cell anisotropic growth, division patterns, and overall organ shape) were highly expressed in *fs8.1* NIL; meanwhile, *IQD10/21*, *CYP724A1* (encoding a cytochrome P450 enzyme which plays a role in brassinosteroid biosynthesis), *IAA19/29* (encoding AUX/IAAs which participates in auxin signal transduction), *PIN3* (encoding an auxin polar transporter), *NCED1*, *CYP704A2* (encoding a cytochrome P450 enzyme whose homolog is associated with seed size in rice (Tang et al., 2016)), *CEPR2* (encoding a receptor protein-tyrosine kinase which mediates nitrate uptake signaling) and *CKX7* (encoding a cytokinin oxidase which is involved in cytokinin degradation) were up-regulated in NILs harboring *fs8.1*; in contrast, expression of *NR2* (encoding a nitrate reductase which takes part in nitrogen assimilation), *NRT1.1* (encoding a dual-affinity nitrate transporter), *IAA36*, *CKI7* (encoding a cyclin-dependent kinase inhibitor 7 which is involved in cell cycle control), *GIF3* (encoding a GRF1-interacting factor whose homolog controls seed size in Arabidopsis), *GA2ox2* (encoding a gibberellin 2-oxidase which plays a role in gibberellin degradation), *WLIM1* (encoding a F-actin binding protein containing LIM domain which takes part in actin cytoskeleton remodeling (Thomas et al., 2007)) was reduced in the NILs harboring *fs8.1*; as to *CYCD3* (encoding a cyclin-D3-3 which is involved in the switch from cell proliferation to the final stages of differentiation), *CYP85A1* (encoding a BR6ox enzyme which is involved in brassinosteroid biosynthesis), *IAA1*, *CYP707A1* (encoding an abscisic acid 8’-hydroxylase which catalyzes the degradation of ABA), they highly expressed in NILs *s/f*, *o/f* and *s/o/f*; expression of *SWEET14* (encoding a bidirectional sugar transporter protein), *2ODD* (encoding a 2-oxoglutarate-dependent dioxygenases which is involved in gibberellin biosynthesis), *DWF1* (encoding a steroid synthetase which plays a role in brassinosteroid biosynthesis), *NRT1.11*, *GA2ox2*, *OFP10*, *WLIM1*, *2ODD* was obviously higher in WT NIL (**Figure 5B**). In regard to left genes, *PIN5* was up-regulated in NIL *fs8.1* and *s/o*; *GRP6* was activated in NILs *fs8.1* and *s/f*; *VI2* (encoding a vacuolar invertase 2 protein which is involved in sugar metabolism), *STP1* (encoding a sugar transporter protein) and *NRT1.1* clearly highly expressed in *o/f* NIL (**Figure 5B**)

In the Venn diagram of NILs *sun*, *ovate* and *s/o*, 135, 75 and 192 DEGs were unique to NILs *sun*, *ovate* and *s/o*, respectively; meanwhile, 31, 51 and 30 DEGs were only shared between NILs *sun* and *ovate*, NILs *sun* and *s/o*, and NILs *ovate* and *s/o*, respectively; in addition, 38 DEGs were shared by the three NILs (**Figure 6A**). According to their annotations, 14 interested DEGs were screened out and 10 were unique to *s/o* NILs. Among the 10 DEGs, *CEPD2* (encoding a member of the CC-type glutaredoxin family which is induced by nitrogen starvation) and *NRT1.1/2/2.4* were obviously up-regulated in the *s/o* NIL; meanwhile, an abscisic acid receptor gene *PYR1-L* was clearly down-regulated in *s/o* NIL but expressed higher in NILs WT, *sun*, *ovate* and *s/o/f*; as to *CKX3*, it highly expressed in NILs *s/f*, *o/f* and *s/o/f*; expression of *SCL3*, a gene involved in GA signaling, was higher in NILs WT and *sun*; as to *GA20ox1*, it was obviously up-regulated in *fs8.1* NIL, but was clearly down-regulated in *s/o* NIL; in regard to the expression of *IAA1* and *2*, the highest levels were recorded in NILs *s/o/f* and *s/o*, respectively (**Figure 6A**).

In the Venn diagram of NILs *sun*, *fs8.1* and *s/f*, 119, 289 and 399 DEGs were unique to NILs *sun*, *fs8.1* and *s/f*, respectively; meanwhile, 29, 67 and 201 DEGs were only shared between NILs *sun* and *fs8.1*, NILs *sun* and *s/f*, and NILs *fs8.1* and *s/f*, respectively; in addition, 40 DEGs were shared by the three NILs (**Figure 6B**). Based on the putative functions, 45 interested DEGs were screened out, of which 26 were unique to *s/f* NIL (**Figure 6B**). Among the 26 DEGs, *GH3.1* (encoding a *Grethchen Hagen 3* family protein which takes part in inactivation of IAA), *GH3-10*, *GA2ox2*, *CKX1/3/7*, *IPT1* (encoding an isopentenyl transferase which is involved in trans-zeatin biosynthesis), *SWEET1*, *SUS4* (encoding a sucrose synthase protein), *CYCD3.1/6.1*, *CYP78A10*, *IQD6* and *OFP13* were up-regulated in NILs *s/f*, *o/f* and *s/o/f*; in contrast, *CID1L2* and *CAR4* were down-regulated in the above-mentioned NILs; expression of *SAUR50*, encoding a SAUR-like auxin-responsive protein, was increased in NILs harboring *fs8.1*, while, expression of *WLIM1-L* was decreased in the same NILs; expression of *GH3.2*, *GAST1-L* and *IQD19* was enhanced in *sun* NIL, but was repressed in *s/f* NIL; as to *CYCB2.4* and *ATN* (encoding a microtubule-binding protein), their expression levels peaked in *s/f* NIL, and meanwhile, the expression of *SAUR71* peaked in *fs8.1* NIL; in regard to *2ODD*, its expression was up-regulated in NILs WT and *s/o* (**Figure 6B**).

In the Venn diagram of NILs *ovate*, *fs8.1* and *o/f*, 88, 320 and 358 DEGs were unique to NILs *sun*, *fs8.1* and *s/f*, respectively; meanwhile, 26, 32 and 185 DEGs were only shared between NILs *ovate* and *fs8.1*, NILs *ovate* and *o/f*, and NILs *fs8.1* and *o/f*, respectively; in addition, 28 DEGs were shared by the three NILs (**Figure 6C**). Thirty-eight interested DEGs were screened out of the above-mentioned DEGs, among which *GH3-10*, *IAA3*, *GA2ox1*, *CKX1/3*, *SWEET1-L*, *SUS4*, *CYCD6.1*, *CYP78A10* and *CML25* were up-regulated in NILs *s/f*, *o/f* and *s/o/f*, however, *CAR4* was down-regulated in the same NIL; expression *IAA2* was increased in NILs *s/o*, *o/f* and *s/o/f*; *SAUR50*, *CKX7* and *OFP13* were highly expressed in NILs harboring *fs8.1*, meanwhile, expression of *SAUR70* peaked in *fs8.1* NIL; expression of *GIF1* and *TUB3* (encoding tubulin beta chain 3) was increased in NILs *sun*, *fs8.1*, *s/f*, *o/f* and *s/o/f*; as to the *CML3/8/15/22/24*, they all highly expressed in NILs *o/f* and *s/o/f* (**Figure 6C**).

In order to clarify the epistatic interaction among *sun*, *ovate* and *fs8.1* in the triple NIL, Venn diagram analysis was also performed with NILs *s/o*, *s/f*, *o/f* and *s/o/f*. The results showed that 400 DEGs were unique to *s/o/f* NIL; 41, 134 and 71 DEGs were only shared between NILs *s/o/f* and *s/o*, NILs *s/o/f* and *s/f*, and NILs *s/o/f* and *o/f*, respectively; 27, 308 and 15 DEGs were only shared by NILs *s/o/f*, *s/o* and *s/f*, NILs *s/o/f*, *s/f* and *o/f*, and NILs *s/o/f*, *s/o* and *o/f*, respectively; 55 DEGs were shared by all four NILs (**Figure 5C**). Sixty interested DEGs were screened out from the above-mentioned DEGs, of which 21 were unique to *s/o/f* NIL (**Figure 5C**). Among the 21 DEGs, the highest expression levels of *YUCCA6* (encoding an indole-3-pyruvate monooxygenase), *GH3.1/3.6*, *GA2ox8-L*, *DWF2*, *CYP85A1*, *CYCA3.2*, *GRF3-L*, *WRKY31*, *dUTPase* (encoding a deoxyuridine 5’-triphosphate nucleotidohydrolase whose homologous positively regulates grain length in rice (Utsunomiya et al., 2011)), *NRT1.11/1.2*, *MAP65-6* and *CML35/60D* were all in the *s/o/f* NIL; in contrast, for the left genes, including *GA2ox1*, *GRP*, *GRP13*, *SPSB* (encoding a sucrose-phosphate synthase B) and *OFP10*, the lowest expression levels were all discovered in the *s/o/f* NIL (**Figure 5C**).

In another aspect, when considering the expression patterns of all the interested DEGs, they could be classified into 17 groups. Group I to XVII mainly represented the DEGs that highly expressed in NILs harboring *fs8.1*; only in *fs8.1* NIL; in NILs *s/f* and *s/o/f*; in NILs *s/f*, *o/f* and *s/o/f*; only in *s/o/f* NIL; in NILs WT, *ovate* and *fs8.1*; in NILs WT and *sun*; in NILs WT and *fs8.1*; in NILs WT, *ovate*, *fs8.1* and *s/o*; in NILs WT, *sun* and *fs8.1*; in NILs WT and *ovate*; in NILs WT, *ovate* and *s/o*; in NILs WT, *sun*, *ovate* and *s/o*; in NILs WT, *sun*, *ovate*, *fs8.1* and *s/o*; only in *sun*; only in *s/o*; and DEGs that lowly expressed in *fs8.1* NIL, respectively (**Figure 6D**).

Besides what is mentioned above, for further understanding the mechanism of fruit shape loci in the regulation of seed morphogenesis, expression of *SUN* and *OVATE* was screened out from the RNA-seq data of *S. pimpinellifolium* ovaries (Pattison and Csukasi, http://ted.bti.cornell.edu/cgi-bin/TFGD/digital/experiment.cgi?ID=D009) as well as our developing seeds. In the WT NIL, *SUN* barely expressed in anthesis ovules, 4-dpa embryos, 4-dpa endosperm, 4-dpa seed coat and 4-dpa funiculus but highly expressed in 8-dpa seeds (**Figures S8A, B**). While, *OVATE* highly expressed in anthesis ovules and 4-dpa funiculus but barely expressed in embryos, endosperm and seed coat at 4 dpa (**Figures S8C, D**).

In order to verify the expression patterns of some important genes, qRT-PCR was conducted and the results were consistent with those observed in the RNA-seq (**Supplementary Figure S9**).

## 4 Discussion

### 4.1 Fruit shape loci played different roles in the regulation of tomato seed shape and size than they did in the control of fruit morphology

Since *sun*, *ovate* and *fs8.1* have been fine-mapped in tomato, their functions in the regulation of plant organ morphogenesis have been well studied in Arabidopsis, rice and a number of horticultural crops (Wang et al., 2011; van der Knaap et al., 2014; Burstenbinder et al., 2017b; Pan et al., 2017; Dou et al., 2018; Lazzaro et al., 2018; Yang et al., 2018a). *sun* and some gain-of-function mutations of *IQDs* regulate organ shape through simultaneously promoting and inhibiting cell proliferation in the longitudinal direction and cross direction, respectively (Wu et al., 2011; Yang et al., 2018a). *ovate* and the loss-of-function mutations of *OFPs* elongate fruits and tubers mainly through increasing cell number in proximal-distal direction but decreasing cell number in medio-lateral direction, especially in the proximal part (Wu, 2015; van der Knaap and Ostergaard, 2018). As to *fs8.1*, it enlarges fruit shape index and size mainly through enhancing cell division along the longitudinal axis (Sun et al., 2015). However, in this study, totally different regulatory patterns of the fruit shape loci were discovered in the control of seed shape and size. In particular, *sun* significantly reduced SSI through decreasing seed length, which could be attributed to the inhibition of seed coat cell proliferation in proximal-distal direction (**Figures 2A, D–F**; **Tables 1**, **3**). This pattern was not only different from that in fruit, but also in contrast with that of *GSE5* in rice (Duan et al., 2017). *GSE5* encodes an IQD which is closely related to Arabidopsis IQD25-27. Up-regulation of *GSE5* led to increased SSI through increased cell proliferation in spikelet hulls, whereas, down-regulation of its expression resulted in wider grains (Duan et al., 2017). The difference between *sun* and rice *GSE5* mutations may be attributed to the different seed structures between the dicotyledon and monocotyledon. In regard to *ovate*, it down-regulates SSI through decreasing seed length but at the same increasing seed width (**Figures 2A, D–F**; **Table 1**). Since seed coat cell number was not significantly changed and the total cell number of embryo was increased, the decreased seed length of *ovate* NIL could be attributed to the down-regulation of embryo crimp ratio (**Figure 2D**; **Table 3**). As to the seed width, now that no significant difference was found in seed coat cell layer, it could be presumed that the increased seed width of *ovate* NIL was attributed to additive effect of the increased cavity size and the increased embryo cell number in medio-lateral direction (**Figure 2E**; **Table 3**). In the *fs8.1* NIL, the reduced SSI was mainly due to the significantly increased seed width, which was high-likely caused by the additive effect of the increased trends of seed coat cell layer and embryo cell number in medio-lateral direction (**Figures 2D–F**; **Table 3**). Besides the single loci, double loci interaction and triple loci interaction also played critical roles in impacting seed length, width and shape index (**Table 2**), which was also not fully consistent with what has been observed in the regulation of fruit shape (Wu et al., 2015). In the previous investigation of same NILs, only interactions of *sun*×*ovate* and *sun*×*fs8.1* showed significant effects on fruit shape index (Wu et al., 2015), whereas, in this study, interactions of *sun*×*fs8.1* and *sun*×*ovate*×*fs8.1* significantly impacted SSI (**Table 2**), which further suggested the different patterns and mechanisms underlying the regulation of seed shape by fruit shape loci.

In another aspect, significant decreases of seed size and weight were found in the double and triple NILs (**Figures 2B, C**) and this phenomenon was never observed in fruits of the same NILs (Wu et al., 2015), which also indicated the mechanisms of fruit shape loci in the regulation of seed development was different from those in fruit. Based on the histological analysis, *s*/*o* and *s*/*o*/*f* significantly decreased seed coat cell number and embryo crimp ratio, which led to the down-regulation of seed coat size and embryo size, and finally resulted in smaller seeds (**Figures 2A, B**; **Table 3**). *o*/*f* significantly decreased seed size mainly through reducing the seed coat size, which could be attributed to the significant decrease of cell number in seed coat (**Figures 2A, B**; **Table 3**). *s*/*f* had the smallest seed size which was caused by the significant reduction of seed coat size and embryo size. The decreased embryo size in *s*/*f* NIL was mainly attributed to significant down-regulation of cell number in cotyledon and hypocotyl. As to the seed coat, since cell number was significantly decreased, logically the reduction of seed coat cell number should be the major reason for the decrease of seed coat size (**Figures 2A, B**; **Table 3**).

Taking together, although *sun*, *ovate* and *fs8.1* still mainly acted as cell number regulators in this study, they played obviously different roles in the regulation of tomato seed shape and size than they did in the control of fruit development.

### 4.2 Fruit shape loci regulated tomato seed shape and size mainly through impacting phytohormone-, sugar- and cytoskeleton-related pathways

For further understanding the mechanisms underlying the morphological and histological variations of seeds induced by the fruit shape loci, phytohormones and transcriptomes of the developing seeds were investigated, and phytohormone-, sugar- and cytoskeleton-related pathways were discovered to be significantly impacted by the fruit shape loci.

Phytohormones are always considered as critical regulators of seed development. GA, CK and BR generally play positive roles in the regulation of seed size (Werner et al., 2003; Jiang et al., 2013; Fang et al., 2016; Hu et al., 2017; Zhou et al., 2017; Gan et al., 2022), whereas, ABA has been proved to be negatively associated with seed size (Cheng et al., 2014; Wang et al., 2021). As to auxin, its local accumulation and polar transportation also take part in the control of seed development (Liu et al., 2015b). In this study, *sun*, *ovate* and *fs8.1* showed distinct effects on adjusting the phytohormone-related pathways. Particularly, *sun* only significantly reduced the accumulation of BR by down-regulating the expression of the BR biosynthesis gene *DWF1* (**Figure 4D**; **Figure 5B**). While, *ovate* significantly decreased the GA level through repressing the expression of *2ODD* (**Figure 4D**; **Figure 5B**), a gene taking part in GA biosynthesis (Huang et al., 2010). Additionally, expression of *PIN5*, a gene encoding an auxin polar transporter, was also decreased by *ovate* (**Figure 5B**), which could impact the auxin polar transportation and local auxin gradients in developing seeds, and may finally change seed shape and size. As to *fs8.1*, it showed a pleiotropic effect on phytohormones that GA, ABA and BR contents were significantly increased, whereas, the CK level was obviously decreased (**Figures 4B–E**). Consistently, in *fs8.1* NIL, expression of *GA2ox* and *CYP707A1* was down-regulated, whereas, expression of *NCED1*, *NCED3*, *CYP724A1* and *CKX* was up-regulated (**Figure 5B**). The impacts of the fruit shape loci on the phytohormone level were also discovered in the developing flowers at 9 dba (days before anthesis) (Wu et al., 2015). In young flower buds, *sun* significantly increased JA (jasmonic acid) and JA-Ile (isoleucine conjugated-JA) levels in the absence of the *fs8.1*; *ovate* had an impact on iP (isopentenyladenine, a kind of CK) accumulation; *fs8.1* not only tend to reduce IAA and increase ABA in 9-dba flower buds but also inhibited TRA (tryptamine, an auxin precursor) accumulation in anthesis ovaries and 5-dpa fruits (Sun et al., 2015; Wu et al., 2015). Therefore, it can be presumed that phytohormones are high-likely the targets of the three fruit shape loci, however, the regulatory mechanisms of different loci or the same locus in different organs are not exactly the same. Interestingly, the increased ABA level was observed in both the developing seeds (**Figure 4E**) and young flower buds (Wu et al., 2015) harboring *fs8.1*, indicating this may be a conserved mechanism underlying the regulation of organ morphology by *fs8.1*.

Additionally, interactions were discovered among different loci at the hormone level. In *sun* background, in addition to the decreased CK level, *fs8.1* also significantly reduced IAA and GA levels but did not impact ABA level (**Figures 4A–C**). Consistent with that, in the *s/f* NIL, *GH3.1*, *GA2ox* and *GKX1/3/7* were up-regulated, whereas, *2ODD* was down-regulated (**Figure 6B**). However, in *ovate* background, *fs8.1* only reduced IAA and CK levels by up-regulating the expression of *GH3-10* and *CKX1/3/7* (**Figures 4A, C**; **Figure 6C**). Thus, it can be deduced that interactions of *sun*×*fs8.1* and *ovate*×*fs8.1* enhanced the impact of *fs8.1* on the IAA accumulation but eliminated the positive effect of *fs8.1* on ABA and GA accumulations. In another aspect, interaction between *sun* and *ovate* significantly reduced GA and CK levels through simultaneously down-regulating the expression of *GA20ox1* but up-regulating the expression of *CKX3*, respectively (**Figure 4B, C**; **Figure 6A**), which has never been observed in NILs harboring *sun* or *ovate* alone (**Figure 4**; **Figure 5B**), indicating new regulatory pathways were established by the interaction. As to the interaction of *sun*×*ovate*×*fs8.1*, it inherited most of the effects of the interactions of the double NILs, except for that of ABA, which was significantly increased in *s/o/f* NIL (**Figures 4A–D**). Interactions among *sun*, *ovate* and *fs8.1* were also observed in 9-dba young flower buds in the regulation of iP, SA, JA and JA-Ile but not in that of IAA and ABA (Wu et al., 2015), indicating the interaction patterns among the three fruit shape loci may be different in the regulation of different organs.

Sugar metabolism and transport also play an important part in the regulation of seed development (Xu et al., 2019; Liao et al., 2020; Wang et al., 2020). In the developing seeds, *sun* significantly down-regulated the expression of *VI2* and *SWEET* (**Figure 5B**), which could influence the energy transportation from the source to the sink and may repress cell number by inhibiting cell proliferation. Evidences supported the above-mentioned hypothesis came from studies in rice and soybean that a mutation of *OsVIN2*, a homolog of *VI2*, resulted in decreased grain size by altering sugar metabolism (Xu et al., 2019), meanwhile, *GmSWEET10a* was confirmed to be positively correlated with seed size (Wang et al., 2020). Similar to what happened in *sun* NIL, expression of *SWEET* and *VI2* was also down-regulated in *ovate* NIL (**Figure 5B**). Furthermore, expression of *STP1*, which belongs to the monosaccharide transporter (*MST*) gene family and plays a key role in maintaining source/sink characteristics (Liu et al., 2021), was also repressed (**Figure 5B**), suggesting *ovate* may have a stronger impact on the source-sink relationship of seed than *sun*. Different from what is mentioned above, *fs8.1* increased the expression of *CWI1* (**Figure 5B**). This gene encodes a cell wall invertase which is involved in the hydrolyzation of the sucrose into glucose and fructose and is positively corelated with seed size (Liao et al., 2020). Thus, it can be presumed that the smaller seeds in NILs *sun* and *ovate* could be partially attributed to the impaired source-sink relationship, whereas, the larger seeds in *fs8.1* NIL are high-likely partially ascribed to the activated hydrolyzation of the sucrose.

Interactions were also discovered in the sugar-related pathways. Compared with that in *sun*, the inhibition of sugar transportation and degradation was relieved in *s/f* NIL, because expression of *SWEET14* and *VI2* was no longer repressed and the transcriptional levels of *SWEET-like* and *SUS4* were up-regulated (**Figure 6B**). *SUS4* encodes a sucrose synthase (SuSy) and is capable of catalyzing the first degradative step in sucrose utilization (Yao et al., 2020). Similar to what happed in *s/f* NIL, the inhibition of sugar transportation and degradation was also relieved in o*/f* NIL, which was caused, on the one hand, by the up-regulation of *SWEET1* and *SUS4*, and on the other hand, by relieving the repression of the expression of *SWEET14* and *VI2* (**Figure 6C**). These phenomena suggested that a common interaction pattern may be shared by *sun*×*fs8.1* and *ovate*×*fs8.1* in the sugar transportation and degradation during seed development. As to the interaction between *sun* and *ovate*, it is worth mentioning that although *sun* and *ovate* alone inhibited the expression of sugar transportation- and degradation-related genes, those genes were not significantly altered in *s/o* NIL, indicating that the interaction of *sun*×*ovate* relieved this inhibition. In regard to *s/o/f* NIL, only *SPSB*, a gene encoding a sucrose-phosphate synthase, was identified in the *s/o/f* unique DEGs (**Figure 5C**), indicating the interaction of *sun*×*ovate*×*fs8.1* almost eliminated all the above-mentioned effects of the fruit shape loci on sugar metabolism and transport.

Many experimental evidences support that the biological functions of *SUN* and *OVATE* are based on their roles in the rearrangement of the cytoskeleton (Abel et al., 2013; van der Knaap et al., 2014; Burstenbinder et al., 2017b; Lazzaro et al., 2018; Wendrich et al., 2018; Wu et al., 2018). In this study, *sun* reduced the expression of *WLIM1* (*Solyc03g114000*) (**Figure 5B**), which encodes a F-actin binding protein and is involved in actin cytoskeleton remodeling (Thomas et al., 2007). Interestingly, in the developing flower buds, expression of another *WLIM1* gene was also impacted by *sun* (Wang et al., 2019), suggesting this class of gene may be the target of *sun* in the control of tomato fruit and seed morphology. Similarly, *ovate* also down-regulated the expression of *WLIM1* (**Figure 5B**), on the one hand, indicating the WLIM1-related pathway was also the target of *ovate* in the regulation of seed morphology, on the other hand, confirming that *sun* and *ovate* share more pathways in this process. It is worth noting that another member of the *OFP* gene family was also down-regulated in *ovate* NIL, which may further enhance the effect of *ovate* on the reduction of seed size and shape index. In regard to *fs8.1*, it significantly reduced the expression of *WLIM1* (*Solyc04g077780*) but increased the expression of *MAP70-5* (**Figure 5B**), which encodes a microtubule-associated protein and may regulate fruit shape through interacting with *IQD21* (Bao et al., 2022). Therefore, it can be deduced that members of the *WLIM1* gene family are high-likely the targets of the three tomato fruit loci in the regulation of fruit and seed morphogenesis.

Interactions among different loci also impacted the cytoskeleton-related pathways. The impacts of *sun* and *fs8.1* on the cytoskeleton-related genes may be enhanced by their interaction, because besides the down-regulated *WLIM1* (*Solyc04g077780*), expression of another *WLIM1* (*Solyc12g088320*) was also reduced in the *s/f* NIL, and meanwhile, expression of *ATN*, a gene involved in the identification of the division plane during mitosis, was up-regulated (**Figure 6B**). Similarly, in the *o/f* NIL, expression of *WLIM1* (*Solyc04g077780*) was still down-regulated, however, *TUB3* was up-regulated by the interaction. In contrast, interaction between *sun* and *ovate* eliminated the effect of single loci on cytoskeleton. As to the interaction of *sun*×*ovate*×*fs8.1*, it eliminated the effects of single loci, but at the same, established a new regulatory pathway which consisted of *MAP65-6* and *ROP9*.

Besides what is mentioned above, based on the expression patterns of *SUN* and *OVATE* in WT NIL (**Figure 5B**), it can be presumed that variations at the phytohormone and transcriptomic levels in NILs harboring *sun* could be directly attributed to effects of this locus; whereas, the physiological and transcriptomic variations in NILs carrying *ovate* were probably ascribed to the after effects of the locus or influence of nearby tissue, such as funiculus, since *OVATE* still highly expressed in this tissue at 4 dpa (**Figure S8D**).

Taking together, *sun* impacted tomato seed morphology mainly through reducing BR biosynthesis, inhibiting sugar transport and degradation as well as repressing WLIM1-related cytoskeleton arrangement; meanwhile, *ovate* regulated seed morphology mainly by inhibiting auxin polar transportation as well as GA biosynthesis, sugar transport and degradation, and WLIM1-related cytoskeleton arrangement; as to *fs8.1*, although a decreased CK level and an increased ABA level were observed in the developing seeds of this NIL, the increased GA and BR levels as well as the enhanced source-sink relationship were still able to result in an increased trend of seed size. Interaction between *sun* and *fs8.1* changed seed morphology mainly through decreasing the IAA, GA and CK contents as well as further repressing the WLIM-related cytoskeleton pathway; interaction between *ovate* and *fs8.1* impacted seed morphology mainly through down-regulation of the IAA and CK contents, and repressing WLIM1-related cytoskeleton arrangement; interaction between *sun* and *ovate* changed seed morphology mainly through reducing GA and CK levels; in regard to the interaction of *sun*×*ovate*×*fs8.1*, the effects of the decreased IAA, GA and CK levels may overcome that of increased BR level and finally down-regulated the seed size and shape index together with the rearrangement of cytoskeleton which was caused by the different expression of *MAP65-6* and *ROP9*.

## 5 Conclusion

Based on the data obtained in this study, it can be presumed that *sun* and *ovate* showed potential abilities in decreasing tomato seed size, while, *fs8.1* had a potential ability in increasing this parameter. Interactions between two loci as well as the interaction among three loci all led to significant decrease of seed size. All three fruit shape loci significantly down-regulated SSI, except for *s/f*, which resulted in the reductions in both seed length and width and finally led to a decreased trend of SSI. Histologically, the seed morphological changes were mainly depended on the cell number variations. Transcriptional and physiological analyses discovered that effects of fruit shape loci on seed morphology were high-likely mainly due to the changes of phytohormone-, cytoskeleton- as well as sugar transportation- and degradation-related genes.

## Data availability statement

The datasets presented in this study can be found in online repositories. The names of the repository and accession number can be found below: https://www.ncbi.nlm.nih.gov/bioproject, PRJNA902156.

## Author contributions

LS, HS, and JC designed the experiments; JC, BP, YX, XC, and JJ carried out the morphological, histological and physiological experiments; JC and ZL performed the RNA-seq analysis; JC wrote the first draft of the manuscript; LS revised the manuscript and draw figures. All the authors are contributed to the modification of the manuscript.
