# Supplementary material for: Fruit shape loci sun, ovate, fs8.1 and their interactions affect seed size and shape in tomato
Source: Front Plant Sci. 2023 Jan 12;13:1091639. doi: 10.3389/fpls.2022.1091639 (PMC9879704; doi:10.3389/fpls.2022.1091639)
Supplement: Supplementary file 2 [file DataSheet_1.pdf]

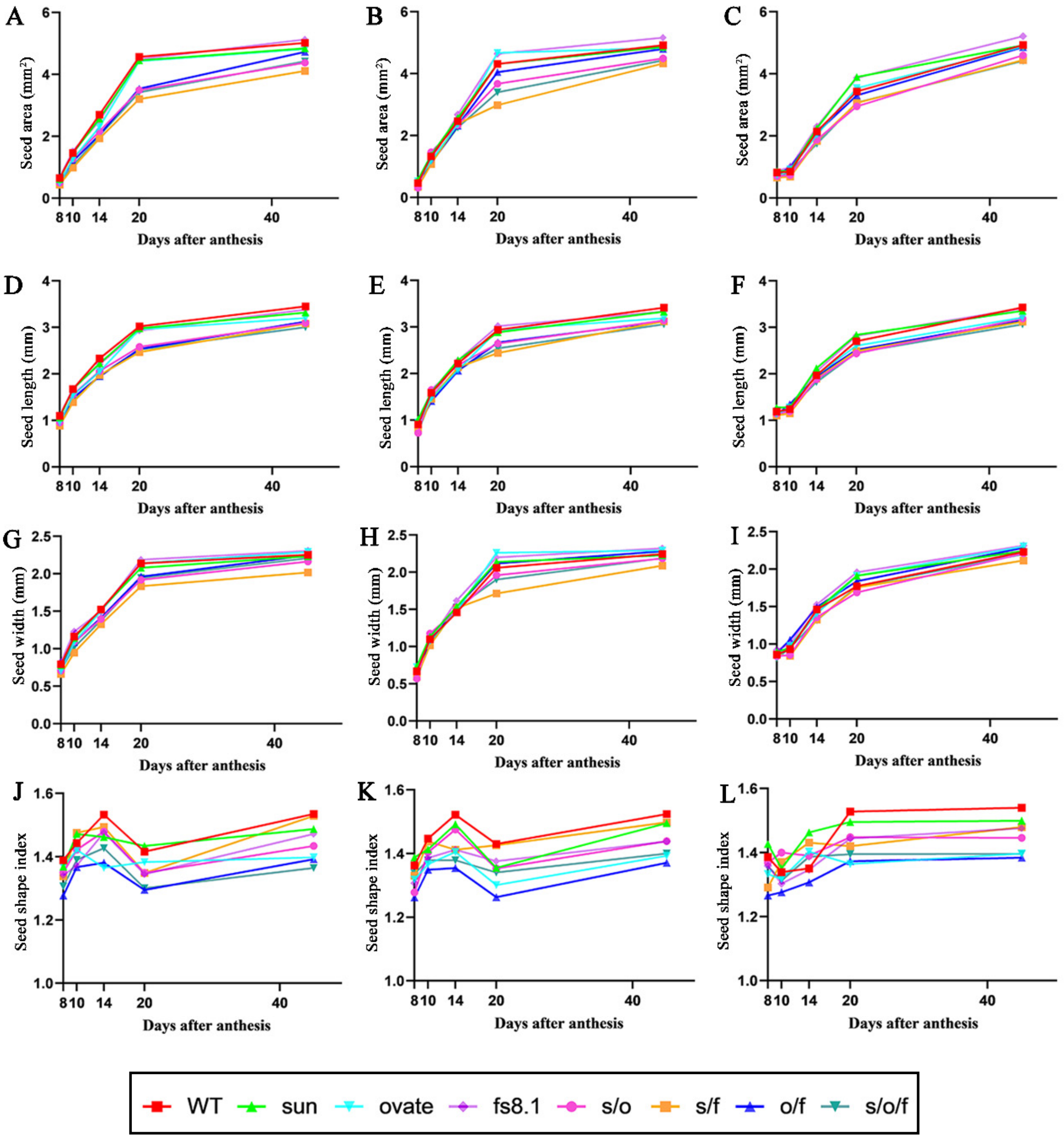

Supplementary Fig. S1. Seed size and shape variation in *sun*, *ovate* and *fs8.1* NILs at different developmental stages. (A-L) three biological repeat of seed area (A-C), length (D-F), width (G-I) and shape index (J-L).

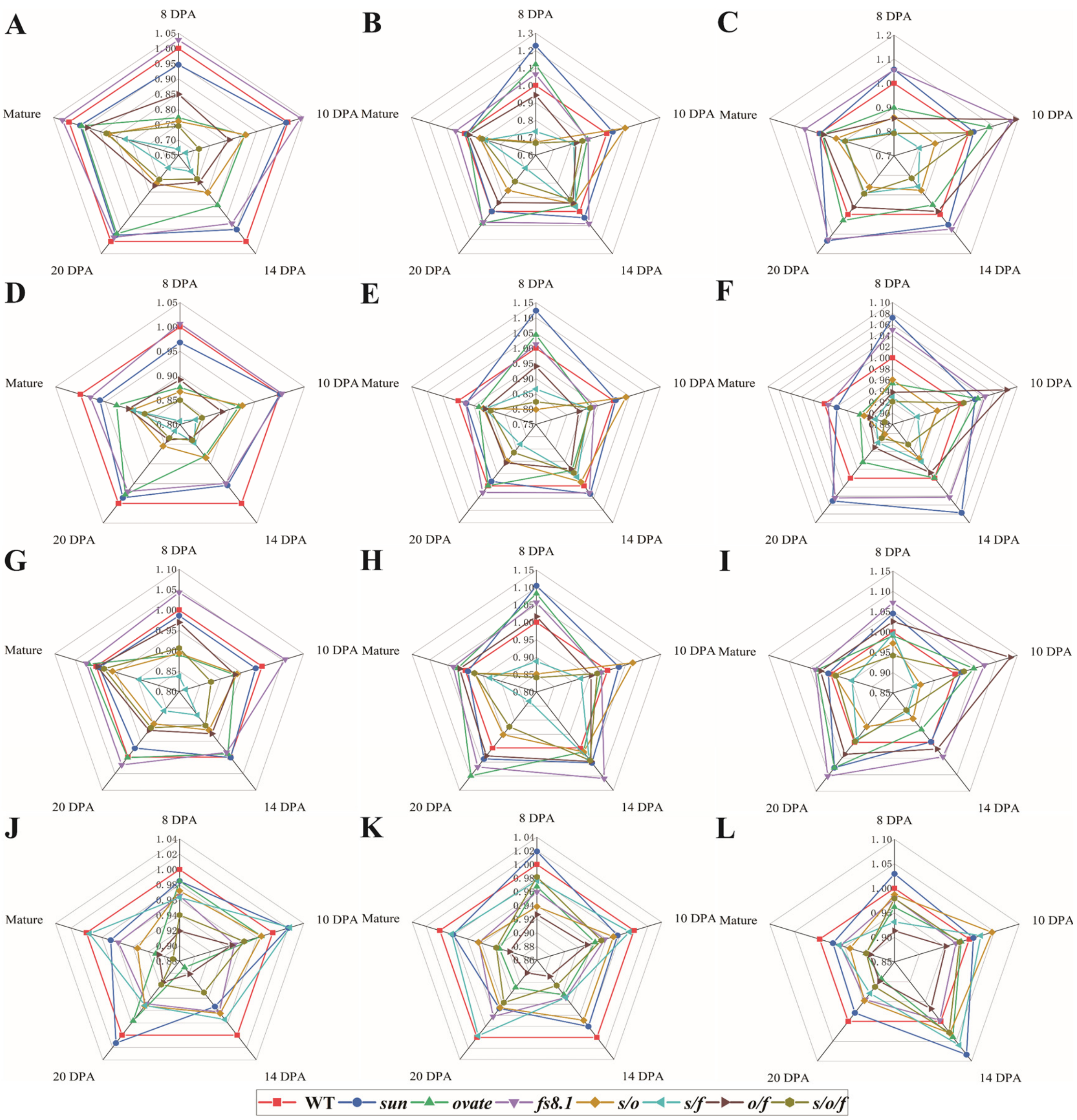

Supplementary Fig. S2. Extent of variation in seed size and shape at various developmental stages relative to WT among *sun*, *ovate* and *fs8.1* NILs. (A-L) Three biological repeats of seed area (A-C), length (D-F), width (G-I) and shape index (J-L).

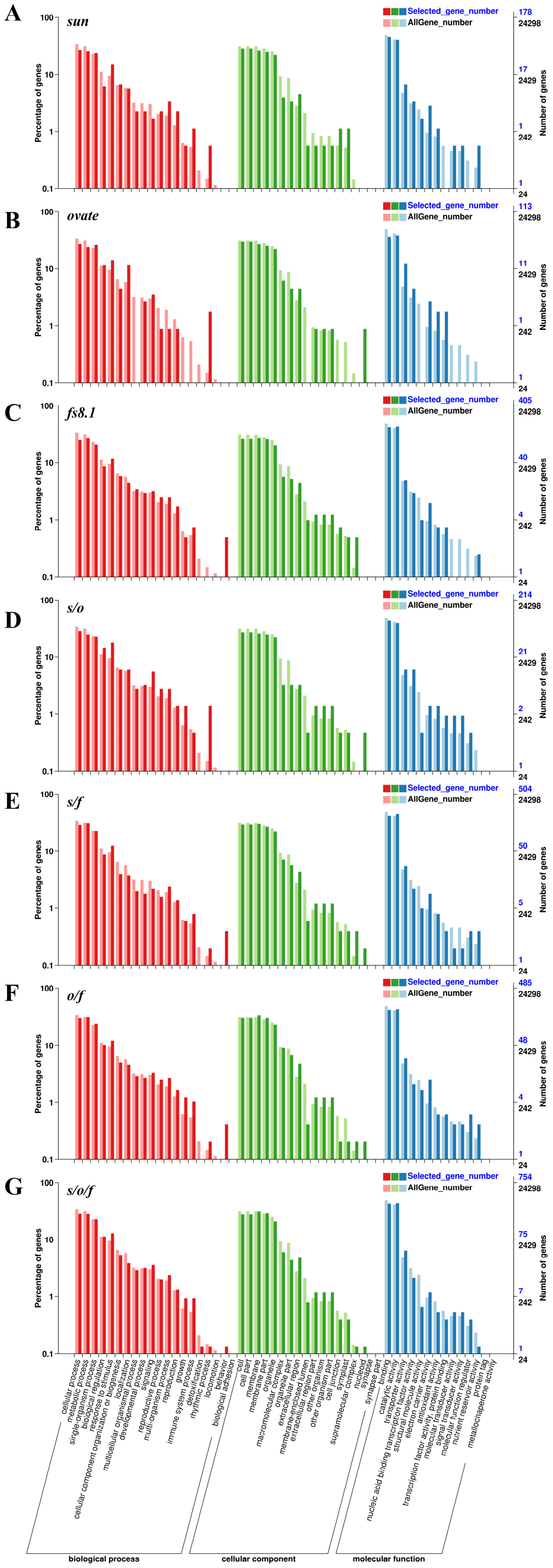

Supplementary Fig. S3. Go enrichment analysis of the DEGs between the WT and *sun* (A), *ovate* (B), *fs8.1* (C), *s/o* (D), *s/f* (E), *o/f* (F) and *s/o/f* (G) NILs at 8 dpa respectively.

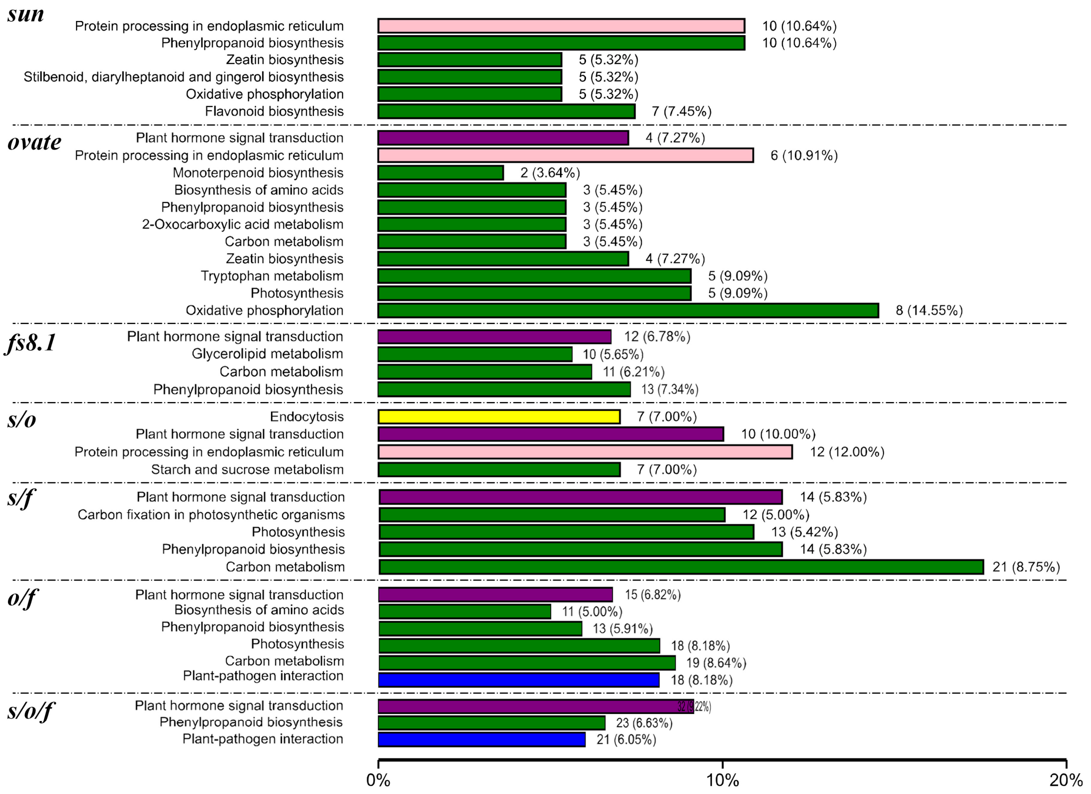

Supplementary Fig. S4. Statistics of KEGG pathway class of the differentially expressed genes (DEGs) in fruit shape NILs.

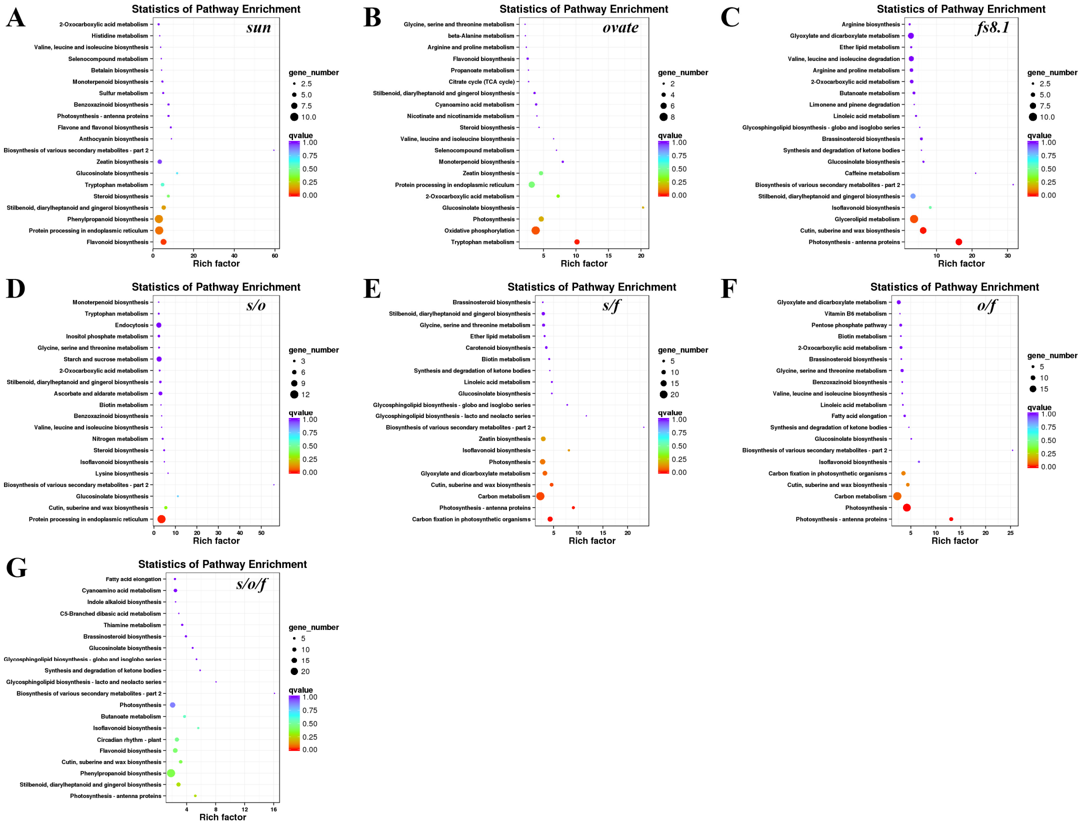

Supplementary Fig. S5. Statistics of KEGG pathway enrichment of the differentially expressed genes (DEGs) in *sun* (A), *ovate* (B), *fs8.1* (C), *s/o* (D), *s/f* (E), *o/f* (F) and *s/o/f* (G) NILs.

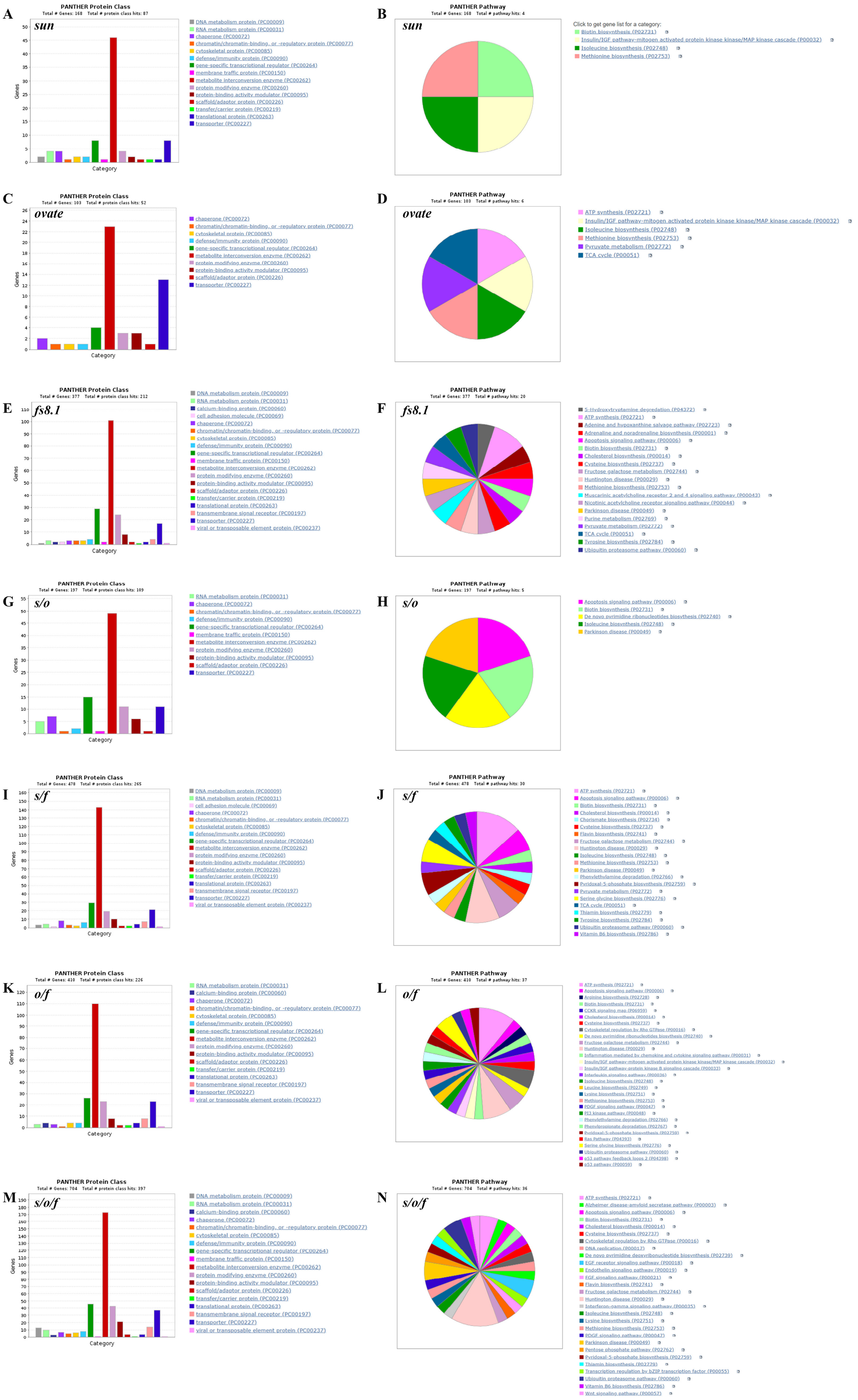

Supplementary Fig. S6. PANTHER protein class and pathway of the differentially expressed genes (DEGs) in *sun* (A, B), *ovate* (C, D), *fs8.1* (E, F), *s/o* (G, H), *s/f* (I, J), *o/f* (K, L) and *s/o/f* (M, N) NILs.

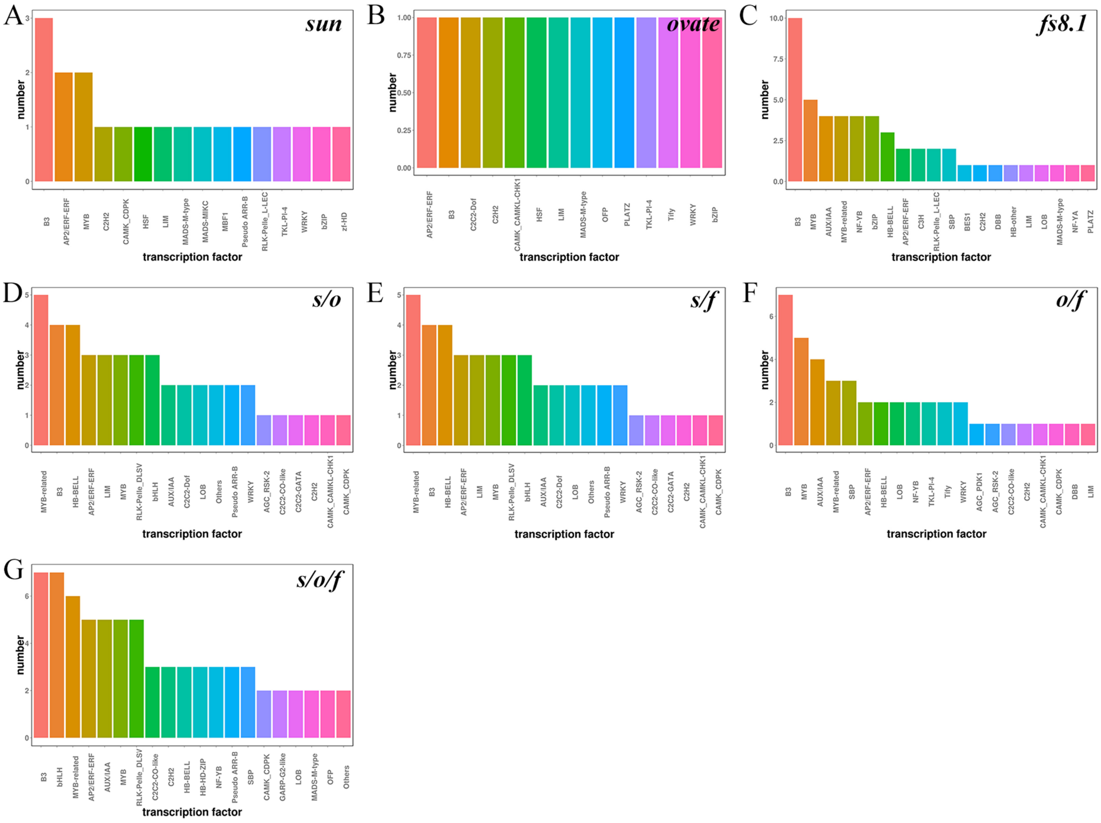

Supplementary Fig. S7. Statistics of transcription factors of the differentially expressed genes (DEGs) in *sun* (A), *ovate* (B), *fs8.1* (C), *s/o* (D), *s/f* (E), *o/f* (F) and *s/o/f* (G) NILs.

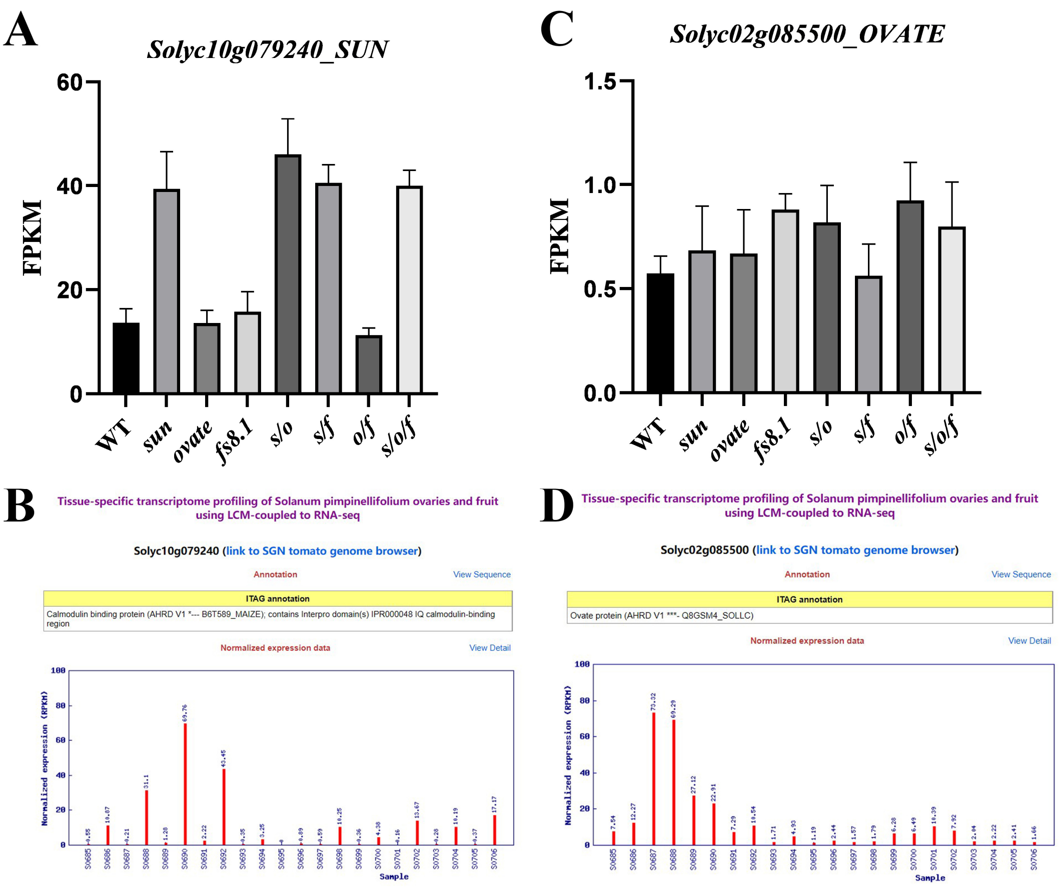

Samples

ID	organism	genotype	description
S0685	tomato	LA1589	LA1589, Ovule 0 dpa (SA1_D0_OV)
S0686	tomato	sun	sun, Ovule 0 dpa (SA2_D0_OV)
S0687	tomato	LA1589	LA1589, Placenta 0 dpa (SA1_D0_PL)
S0688	tomato	sun	sun, Placenta 0 dpa (SA2_D0_PL)
S0689	tomato	LA1589	LA1589, Septum 0 dpa (SA1_D0_SE)
S0690	tomato	sun	sun, Septum 0 dpa (SA2_D0_SE)
S0691	tomato	LA1589	LA1589, Pericarp 0 dpa (SA1_D0_PE)
S0692	tomato	sun	sun, Pericarp 0 dpa (SA2_D0_PE)
S0693	tomato	LA1589	LA1589, Embryo 4 dpa Fruit (SA1_D4_EM)
S0694	tomato	sun	sun, Embryo 4 dpa Fruit (SA2_D4_EM)
S0695	tomato	LA1589	LA1589, Endosperm 4 dpa Fruit (SA1_D4_ENDO)
S0696	tomato	sun	sun, Endosperm 4 dpa Fruit (SA2_D4_ENDO)
S0697	tomato	LA1589	LA1589, Seed Coat 4 dpa Fruit (SA1_D4_SC)
S0698	tomato	sun	sun, Seed Coat 4 dpa Fruit (SA2_D4_SC)
S0699	tomato	LA1589	LA1589, Funiculus 4 dpa Fruit (SA1_D4_FU)
S0700	tomato	sun	sun, Funiculus 4 dpa Fruit (SA2_D4_FU)
S0701	tomato	LA1589	LA1589, Placenta 4 dpa Fruit (SA1_D4_PL)
S0702	tomato	sun	sun, Placenta 4 dpa Fruit (SA2_D4_PL)
S0703	tomato	LA1589	LA1589, Septum 4 dpa Fruit (SA1_D4_SE)
S0704	tomato	sun	sun, Septum 4 dpa Fruit (SA2_D4_SE)
S0705	tomato	LA1589	LA1589, Pericarp 4 dpa Fruit (SA1_D4_PE)
S0706	tomato	sun	sun, Pericarp 4 dpa Fruit (SA2_D4_PE)

Supplementary Fig. S8. Expression of *SUN* and *OVATE* in *S. pimpinellifolium* ovaries (B, D) (Pattison and Csukasi, http://ted.bti.cornell.edu/cgi-bin/TFGD/digital/experiment.cgi?ID=D009) as well as developing seeds (A, C).

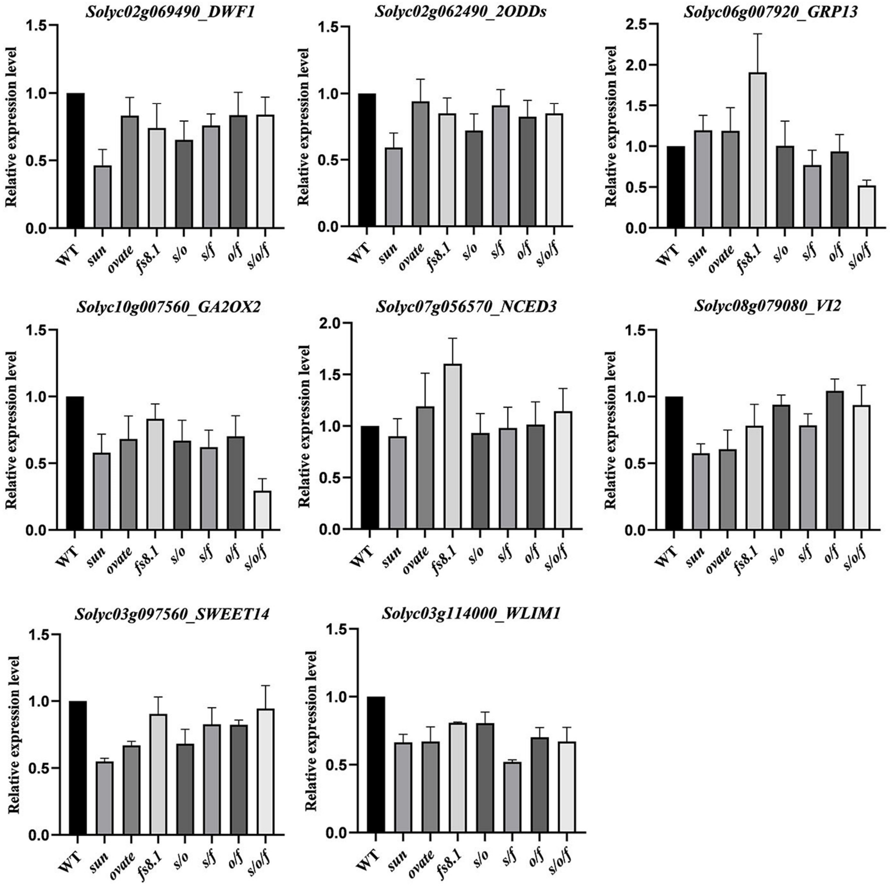

Supplementary Fig. S9. Expression analysis of some important genes in various NILs by using qRT-PCR.

Supplementary Table S1. Correlation analysis of seed size and seed internal structure

attribute	Seed area-1	Seed area-2	Seed area-3	Seed coat area	Cavity area	Embryo area	Embryo length	Embryo crimp ratio
Seed area-1	1							
Seed area-2	0.857**	1						
Seed area-3	0.952**	0.905**	1					
Seed coat area	0.946**	0.922**	0.922**	1				
Cavity area	0.723*	0.748*	0.748*	0.849**	1			
Embryo area	0.874**	0.814*	0.922**	0.837**	0.785*	1		
Embryo length	0.830*	0.732*	0.854**	0.834**	0.897**	0.957**	1	
Embryo crimp ratio	0.31	0.238	0.333	0.216	-0.114	0.072	-0.049	1

** means significant correlation at 0.01 level; * means significant correlation at 0.05 level; correlation analysis were shown by the coefficients computed from the Spearman correlation coefficients in SPSS.
